# Bioinspired self-assembled colloidal collectives drifting in three dimensions underwater

**DOI:** 10.1126/sciadv.adj4201

**Published:** 2023-11-10

**Authors:** Mengmeng Sun, Shihao Yang, Jialin Jiang, Shuai Jiang, Metin Sitti, Li Zhang

**Affiliations:** ^1^ Department of Mechanical and Automation Engineering, The Chinese University of Hong Kong, Hong Kong SAR, China.; ^2^ Physical Intelligence Department, Max Planck Institute for Instelligent Systems, Heisenbergstr. 3, Stuttgart 70569, Germany.; ^3^ Chow Yuk Ho Technology Center for Innovative Medicine, The Chinese University of Hong Kong, Hong Kong SAR, China.; ^4^ Multi-Scale Medical Robotics Center, Hong Kong Science Park, Shatin NT, Hong Kong SAR, China.; ^5^ Department of Surgery, The Chinese University of Hong Kong, Hong Kong SAR, China.; ^6^ CUHK T Stone Robotics Institute, The Chinese University of Hong Kong, Hong Kong SAR, China.

## Abstract

Active matter systems feature a series of unique behaviors, including the emergence of collective self-assembly structures and collective migration. However, realizing collective entities formed by synthetic active matter in spaces without wall-bounded support makes it challenging to perform three-dimensional (3D) locomotion without dispersion. Inspired by the migration mechanism of plankton, we propose a bimodal actuation strategy in the artificial colloidal systems, i.e., combining magnetic and optical fields. The magnetic field triggers the self-assembly of magnetic colloidal particles to form a colloidal collective, maintaining numerous colloids as a dynamically stable entity. The optical field allows the colloidal collectives to generate convective flow through the photothermal effect, enabling them to use fluidic currents for 3D drifting. The collectives can perform 3D locomotion underwater, transit between the water-air interface, and have a controlled motion on the water surface. Our study provides insights into designing smart devices and materials, offering strategies for developing synthetic active matter capable of controllable collective movement in 3D space.

## Teaser

A bimodal actuation strategy is proposed to realize colloidal collectives performing three-dimensional locomotion.

## INTRODUCTION

Nature offers numerous examples of living active matter, which self-assembles into collectives to accomplish complex tasks that surpass individual capabilities, such as bird flocks (*1*) and bacteria colonies (*2*–*4*). Drawing inspiration from natural collectives, many collective systems of artificial active matter [such as colloids (*5*–*8*)] have been developed. Colloids are considered building blocks for materials like atoms which are the bricks of molecules and crystals. Investigating colloidal self-assembly as a means of nanostructure fabrication also holds technological implications. Colloidal structures prepared through self-assembly may find applications in, for example, nanoscale electronics, energy conversion/storage, miniature diagnostic systems, drug delivery, catalysts, and photonic/plasmonic devices (*9*–*13*). The primary means of guiding colloidal assembly include organization on a patterned substrate (*14*), Langmuir-Blodgett assembly (*15*), surfactant-assisted assembly (*16*), assembly in nematic liquid crystals (*17*), assembly in emulsions or inverse emulsions (*18*), assembly in fibers and cells (*19*), and external field-guided particle assembly [e.g., magnetic (*20*–*26*), optical (*27*–*29*), ultrasound (*30*–*32*), and electric fields (*6*, *8*), as well as chemical signals (*33*, *34*)]. However, existing external fields are typically applied to two-dimensional (2D) structures. It will be another step forward if previously unidentified particle manipulation techniques are developed to assemble colloids in 3D arrays.

The controllable 3D motion of colloidal collectives is crucial for assembling colloids in 3D arrays, exploring the physics of self-organization (*35*–*38*), and creating intelligent machine systems (*39*, *40*). Existing strategies for driving the motion of colloidal collectives rely predominantly on physical boundaries, such as liquid-solid and liquid-air interfaces, to introduce spatially asymmetrical interactions (*41*–*44*). Although collectives that depend on the substrate for movement can be maintained as a dynamically stable entity, they are poorly adapted to their environment, e.g., they are unable to detach from the substrate and cross-vertical obstacles that are several times larger than their size (*23*, *43*, *45*–*47*). Therefore, this strong dependence on the presence of a border is a fundamental limitation that impairs colloidal collective maneuverability and their application scenarios. The magnetically actuated helical swimmers can achieve 3D motion, but the swarm formed by them cannot maintain numerous agents as a dynamically stable entity during 3D locomotion (*48*–*50*). Thus, this swarm system based on helically structured particles is not a complete entity, which leads to the risk of its loss and poor targeting during long-distance transportation in 3D space. Combined magnetic and optical fields can achieve tornado-like colloidal swarms but cannot detach from the substrate as a dynamically stable entity (*51*). It is challenging to realize collective entities formed by synthetic active matter in spaces without wall-bounded support to perform 3D locomotion without dispersion.

The diverse collection of organisms found in water (or air) that cannot propel themselves against a current (or wind) is called plankton. Despite some plankton being capable of active motion, they are mainly carried by tides and currents for long-distance migration (*52*, *53*). Plankton are usually thought of as inhabiting water; airborne versions also live part of their lives drifting in the atmosphere. These airborne plankton include plant spores, pollen, and wind-scattered seeds. In summary, the migration mechanism of plankton mainly uses natural flows such as water currents or winds for movement, i.e., drifting with the water or the wind. Inspired by the migration mechanism of plankton swarms in nature, which are carried by tides and currents to perform the 3D collective movement, we propose a method for achieving 3D drifting control of colloidal collectives. We stimulate colloidal collectives to generate a convective flow using a bimodal actuation strategy, combining magnetic and optical fields, which can overcome the limitations of single actuation methods. This approach will enable the controlled collective motion of synthetic active matter in 3D under fluids without dispersion and open possibilities for developing advanced smart devices and materials.

**Fig. 1.**
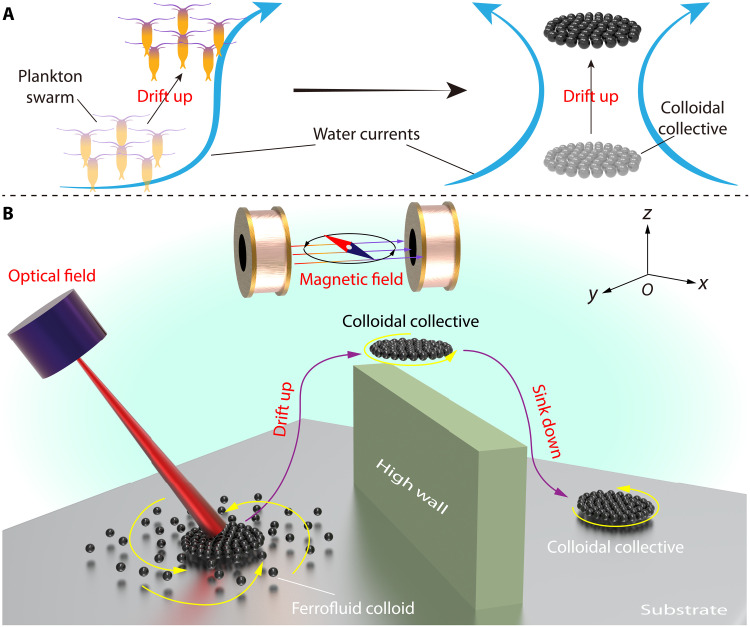
Three-dimensional drifting control of magnetic colloidal collectives. (**A**) The schematic diagram shows the motion mechanism of natural plankton. (**B**) The schematic diagram shows the colloidal collective climbing across a high obstacle under bimodal actuation fields (magnetic and optical fields). First, driven by the tailored rotating magnetic field, the settled ferrofluidic colloids self-assemble into a dynamic stable colloidal collective. Second, the optical field stimulates the colloidal collective to generate convective flow through the photothermal effect, thus allowing the colloidal collective to use currents for 3D drifting motion like the plankton. The proposed colloidal collectives can propel themselves in 3D space, transit between air-water surfaces, and move on the water surface.

In our study, we present an approach for achieving 3D motility of colloidal collectives without dispersion (Fig. 1, A and B). We address the challenges of maintaining collectives from dispersing during 3D drifting, achieving 3D locomotion under fluids while overcoming gravity, and enabling controllable transitions of the collectives through the air-water interface. The colloidal collective consists of ferrofluidic iron colloidal particles with a diameter below 1 μm (see note S1 and fig. S1 for more details) energized by rotating magnetic fields combined with an optical field (see note S2 and fig. S2 for the experimental setup). First, driven by a tailored rotating magnetic field, the settled ferrofluidic colloids self-assemble into a dynamic stable colloidal collective. Next, the focused optical field stimulates the colloidal collective to generate convective flow through the photothermal effect, thus allowing the colloidal collective to use fluid currents for 3D drifting inspired by plankton. Third, we describe methods for water surface to underwater transitions of the colloidal collective and examine their locomotion capabilities on the water surface. These studies culminate in the colloidal collective with 3D motility that adapts to complex environments, representing advances in colloidal robot locomotive capabilities and colloidal self-assembly and manipulation.

## RESULTS

### Bimodal actuation strategy

**Fig. 2.**
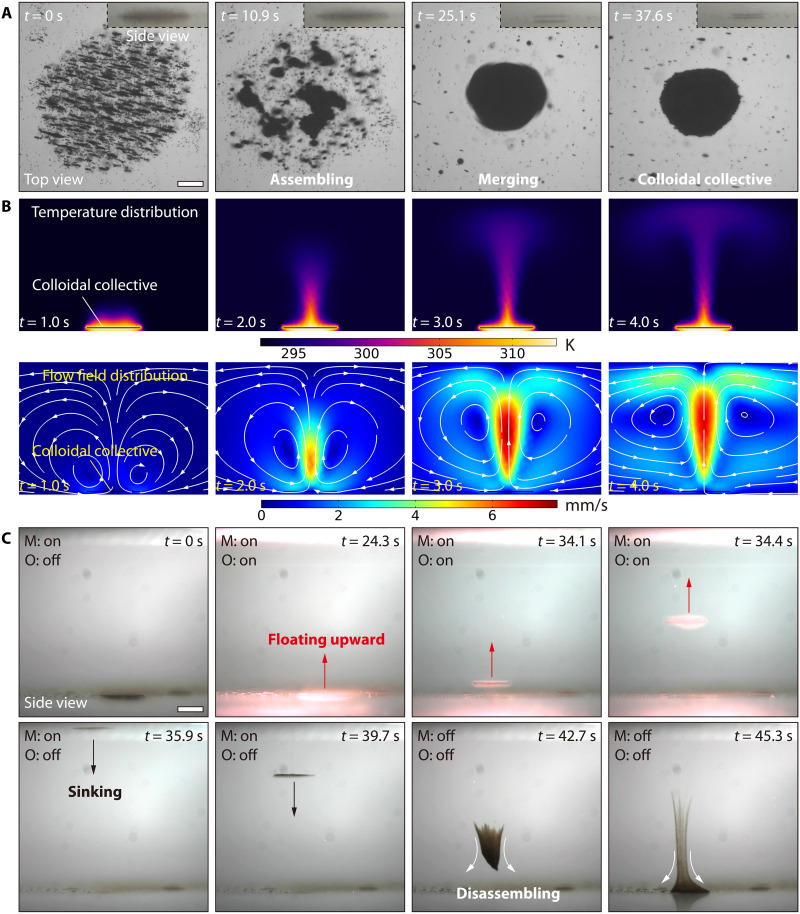
Generation of the upward and downward movements of the colloidal collective. (**A**) Dispersed colloids (<1 μm) dynamically assemble into a colloidal collective inside deionized water upon being energized by the rotating magnetic field (*f*: from 10 to 50 Hz, *B*_m_: 9 mT, θ: 0°). Scale bar, 100 μm. (**B**) Simulation results of the temperature and convective flow velocity distribution around the colloidal collective. The temperature difference between the collective and surrounding fluids (water) is 20 K. The background colors indicate the temperatures and velocities of the surrounding fluid. The white arrows represent the velocity vectors of flow. (**C**) Process in that the colloidal collectives rise and sink. The “M” and “O” labels indicate magnetic (*f*: 50 Hz, *B*_m_: 9 mT, θ: 0°) and optical fields (λ: 808 nm, *P*: 2 W). The red and black arrows indicate the moving directions of the colloidal collective. Scale bar, 1 mm.

We adopt a bimodal actuation strategy of magnetic and optical fields to realize the 3D locomotion of colloidal collectives. The first step involves triggering the formation of the colloidal collective by using a tailored rotating magnetic field that has three adjustable parameters: pitch angle θ, frequency *f*, and strength *B*_m_ (see note S3 and fig. S3 for more details). Initially, in the absence of a magnetic field, the ferrofluidic colloids settle on the tank’s substrate filled with water and exhibit the Brownian motion (at 0 s in Fig. 2A). However, once the colloids are energized by the tailored rotating magnetic field (*f*: 30 Hz, *B*_m_: 9 mT, θ: 0°), they begin to self-assemble, forming small primitive collectives, which are referred to as nonequilibrium colloidal collectives (as illustrated at 10.9 s in Fig. 2A). These primitive collectives continue to increase in size (before 25.1 s in Fig. 2A) and merge. Eventually, a dynamic-equilibrium colloidal collective is generated and maintained (Fig. 2A and movie S1). To analyze the principle of collective generation, two main factors are considered: the magnetic dipole-dipole interactions among the colloids and the hydrodynamic drag force (see note S4 for more details). The governing equation for the velocity of the *i*th colloidal particle is as follows (*20*, *22*, *23*, *26*, *54*–*58*)

$$v_{i}=\frac{F_{i}^{m}+F_{i}^{g}+F_{m}^{\mathrm{rep}}}{6\pi \eta a}+u_{i}$$ (1)

where $F_{i}^{m}$ is the magnetic dipole-dipole interaction force, $F_{i}^{g}$ is the gravitational force, $F_{m}^{\mathrm{rep}}$ is the repulsive force (avoid overlap between colloid-colloid and colloid-wall, and the force is present only when the distance is less than the natural length), and ***u***_*i*_ is the total velocity perturbation of the flow field at *i*th colloidal particle position caused by *N-1* particles. The energized particles assemble into clusters due to time-varying magnetic particle-particle interactions (*54*, *55*). These clusters are attracted to each other and form primitive collectives after several fragmentations and reformation cycles. The primitive collectives draw and merge with neighboring particles, contributing to their growth, as confirmed by simulation (fig. S4 and movie S1). The morphology of the colloidal collective depends on the strength and frequency of the applied magnetic field (fig. S5). By adjusting the pitch angle of the rotating magnetic field, the collective can maintain its integrity and flip over on the substrate, despite gravity (fig. S6 and movie S2). We attribute the magnetic torque that drives the collective’s overturning movement to the tangential component of the dipole magnetic interaction force (see note S5 for more details). The experimental result indicates that the magnetic field can trigger the formation and maintain the dynamic stability of colloidal collectives.

After the colloidal collective is generated, the second step is to use an optical field to induce it to generate controlled convection flows to carry it upward. To determine the feasibility of this strategy, the photothermal effect of the colloidal collective needs to be examined. The dispersed ferrofluid colloidal particles can absorb near-infrared (NIR) light (wavelength λ: 808 nm, power *P*: 2 W) and convert it into heat energy, giving rise to a local temperature gradient (fig. S7). This temperature gradient induces a convective flow in the aquatic environment, which carries the particles upward (fig. S8 and movie S3). Simulation and experimental results indicate that the intensity of the optical field determines the temperature gradient, which affects the velocity of the induced convection (figs. S9 and S10). Under the rotating magnetic field, the dispersed colloidal particles gather into a collective, notably enhancing the photothermal effect. Subsequently, the colloidal collective induces a stronger convective flow under the external NIR optical field, as shown by simulation results in Fig. 2B (see note S7 and movie S4 for more details). The convective flow caused by the photothermal effect overcomes gravity and carries the collective upward without any wall-bounded support (at 24.3 s in Fig. 2C). During the floating-up motion, the magnetic field maintains the colloidal collective, containing numerous individuals as a dynamically stable entity without disintegrating. However, when the NIR optical field is turned off, the colloidal collective cools down, and the hydrodynamic force weakens, causing the collective to sink progressively under gravity (at 35.9 s in Fig. 2C). The relationship between height and time during the floating and sinking process is shown in fig. S11. When the rotating magnetic field is also turned off during the sinking process, the magnetic particle-particle interactions inside the colloidal collective disappear, and the collective begins to disintegrate, with the dispersed particles being deposited on the substrate by gravity (at 42.7 s in Fig. 2C). In addition, the floating-up speed of the colloidal collective can be determined by modulating the power of the external optical field (fig. S12). Moreover, note S8 reveals the dynamic model of colloidal collectives during the floating process, which can be defined as

$$ma=F_{H}+F_{G}+F_{B}$$ (2)

where *m* is the mass of the colloidal collective, ***a*** is the acceleration, ***F***_H_ is the hydrodynamic force from the convection, ***F***_G_ is the gravity, and ***F***_B_ is the buoyancy. On the basis of Eq. 2, the splitting analysis during the rise of the colloidal collective is shown in fig. S13. The phase diagram in fig. S14 shows the magnetic field parameters when the collective is floating without splitting.

### 3D drifting of the colloidal collectives underwater

**Fig. 3.**
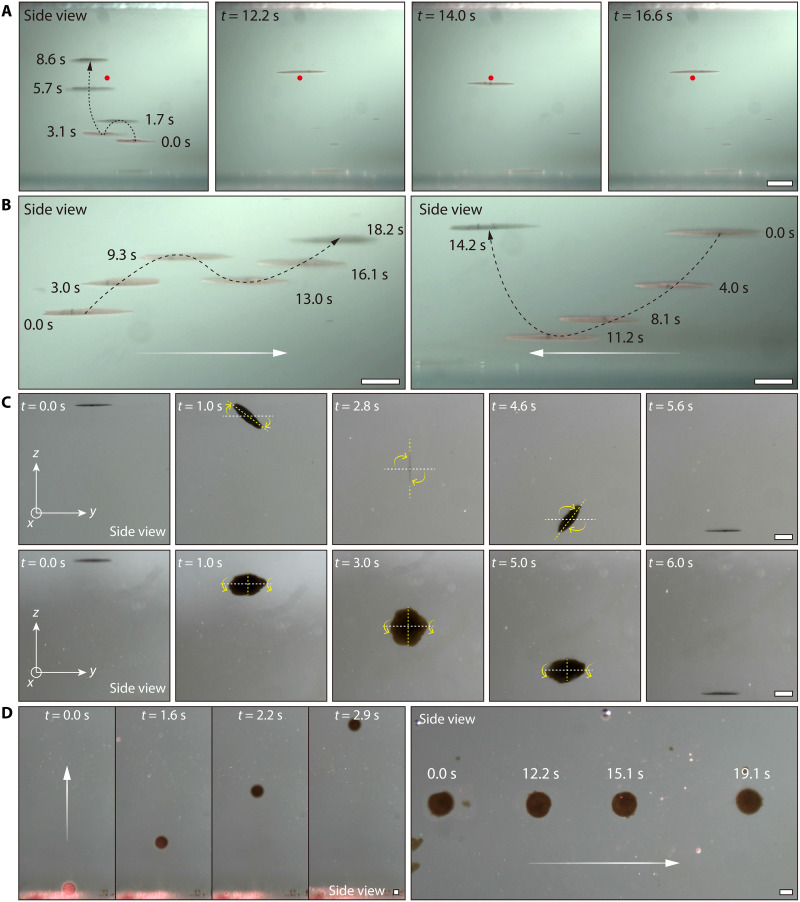
Locomotive characteristics of the colloidal collective underwater. (**A**) Hovering of the colloidal collective. The red spot indicates the desired hovering position. The black dashed lines represent the trajectory of the collective. (**B**) Side-view composite images of the colloidal collective demonstrating directional horizontal locomotion. White arrows indicate the direction of locomotion. (**C**) Process in which the colloidal collective adjusts its posture during the descent. The white dashed line is the horizontal *y* axis. The yellow dotted line is the axis of the colloidal collective. The yellow arrow indicates the overturning direction of the colloidal collective. (**D**) Controlled upward and directional movement of vertical colloidal collective underwater by the induced convective flow. White arrows indicate the direction of locomotion. Magnetic field (*f*: 50 Hz, *B*_m_: 9 mT, θ: 0°) and optical field (λ: 808 nm, *P*: 2 W). [(A) to (D)] Scale bars, 1 mm.

By adjusting the optical field, colloidal collectives can use convection to achieve vertical upward, hovering, and directional horizontal motion. When the optical field uniformly irradiates from directly above, the colloidal collective is at the center of the induced convective flow. The convection pushes the collective vertically upward because the hydrodynamic force is larger than gravity. The convective flow generated by the colloidal collective can be balanced with gravity by continuously controlling the optical field on and off, thus allowing the colloidal collective to hover (movie S5). Figure 3A shows composite images of a 16.6-s hovering floating, and the red dot indicates the setpoint (desired hovering position). For this 16.6-s floating, the maximum deviation of altitude and lateral position are 12 [0.2 body length (BL)] and 36 mm (0.6 BL), respectively (fig. S15A). In addition to controlled hovering, the colloidal collective can also achieve directional locomotion in water by controlling the position of the optical field irradiated onto the surface of the colloidal collective (movie S5). When the colloidal collective is illuminated on the right side, the temperature on the right side is higher than on the left. The induced convection will move from the left side to the right side, thus driving the colloidal collective to the upper right side. Moreover, when the colloidal collective is irradiated on the left side, the induced convective flow will push the colloidal collective to the upper left side. The simulation results in fig. S15B demonstrate the differences in the fluid fields generated when the optical spot irradiates at different positions on the colloidal collective (see note S9 for more details). Controlling the optical field to intermittently illuminate the left or right side of the colloidal collective will direct the colloidal collective to move horizontally to the left or right, respectively. As shown in Fig. 3B, under the intermittent optical field, the colloidal collective moves 5.2 mm to the right side in 18.2 s and 5.0 mm to the left side in 14.2 s, respectively. The relationship between the distance and time of colloidal collectives moving is shown in fig. S15 (C and D).

The magnetic field plays an important role in both preventing the dispersion of colloidal collectives and determining their posture during locomotion. In the next experiment, the optical field is first applied to actuate the colloidal collective to the highest point of the tank. Then, the optical field is turned off, and the colloidal collective sinks under the effect of gravity. Subsequently, the pitch angle of the magnetic field is changed along the *x* axis (from 0° to 180°) and *y* axis (from 0° to 180°), respectively. As a result, the collective will follow the magnetic field for the overall flip motion during the descent (Fig. 3C and movie S6). Notably, the colloidal collective’s flipping motion is less prone to splitting during descent than flipping the entire colloidal collective on the substrate. When the colloidal collective reverses on the substrate, the rotation center is at the end; the rotation center is in the center of the colloid collective when it flips during the falling process. Compared with the flip motion of the colloidal collective on the substrate, the suffered maximum drag force is smaller during the descent (about 0.5 times the flip movement on the substrate), and it is not affected by the role of gravity torque (see note S9 and fig. S16 for more details). Therefore, the magnetic driving torque of the colloidal collective can easily overcome the effect of the sticky resistance torque, which does not easily cause disintegration. Moreover, when the power of the optical field is at 5 W, the vertical colloidal collective obtained by flipping can also achieve floating and directional motion (Fig. 3D). The experimental result indicates that the convective flow generated by the photothermal effect is sufficient to drive the colloidal collective action even when the illuminated area of the collective is minimal. By controlling the optical and magnetic fields, the convection currents induced by colloidal collectives can be modified, thus enabling their multiple motions and posture adjustments underwater.

### Controllable transitions through the air-water interface

**Fig. 4.**
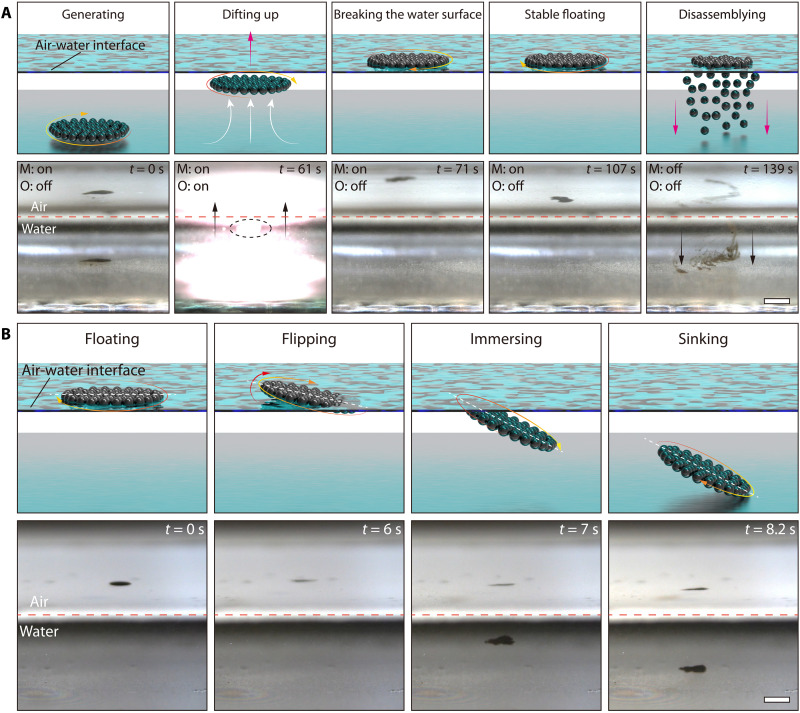
Controllable transition of the colloidal collective through the air-water interface. (**A**) Transition of the colloidal collective from underwater to the water surface. The “M” and “O” labels indicate magnetic (*f*: 50 Hz, *B*_m_: 9 mT, θ: 0°) and optical fields (λ: 808 nm, *P*: 5 W). (**B**) Colloidal collective sinks into water with an inclined posture (*f*: 50 Hz, *B*_m_: 9 mT, θ: from 0° to 20°). [(A) and (B)] Scale bars, 3 mm.

We conduct further investigation into the ability of colloidal collectives to break through the water surface using induced convection flow. However, for colloidal collectives to successfully exit the water, they must first overcome the surface tension of the water. This can be a remarkable challenge for mobile objects at the millimeter scale, as surface tension is highly strong during air-water transitions. Whereas impact forces from large diving objects can easily break the water surface, water entry or exit for millimeter-scale objects is difficult because surface tension is comparable with their weight. The colloidal collectives must overcome surface tension and gravity to enable controllable and repeatable transitions through the water surface. Here, we define transition controllability as the collective’s ability to dive into the water at a desired location and time. The analysis model for colloid collectives when they exit from the water is given by (*59*)

$$F_{B}+F_{H}>F_{S}+F_{G}$$ (3)

where *F*_B_ represents the buoyancy, *F*_H_ represents the hydrodynamic force from the convection, *F*_S_ represents the surface tension, and *F*_G_ represents the gravity (see note S10 and fig. S17A for more details). The previous section has shown that the convection induced under an optical field with a power of 2 W can carry the horizontal colloidal collective to reach below the water surface but not enough to break through the water surface. Since low-strength convection provides insufficient hydrodynamic force for the colloidal collectives to overcome the combined forces of surface tension and gravity, increasing the power of the optical field can induce greater convection. Thus, the hydrodynamic force it provides becomes sufficient for colloidal collectives to break the surface, which is confirmed in Fig. 4A (movie S7). The colloid collective is generated on the substrate under the rotating magnetic field in the initial state (*t* = 0 s). After turning on the optical field, the colloidal collective is carried to the air-water interface bottom (*t* = 61 s). With the continuous irradiation of the optical field, a small number of particles in the collective can adsorb (*56*). These particles serve as attachment points for the rest of the particles in the collective while the field is applied (*t* = 71 s). However, the colloidal collective ruptures into many fragments when crossing the water (fig. S17B). As the colloidal collective suffers from uneven forces during the crossing, and the magnetic dipole force within the collective is not sufficient to maintain stability, it will eventually split into multiple small pieces. Then, driven by the rotating magnetic field, these small collectives begin to regather to form a complete colloidal collective. Under an external magnetic field, the recombined colloidal collective rotates on the water surface without sinking (*t* = 107 s). After turning off the rotating magnetic field, the colloidal collective begins to disintegrate and slowly drop to the bottom of the water. This is because the interparticle magnetic interactions are canceled, and only the adsorbed particles remain at the interface, while the rest fall out due to gravity (*56*).

Once the colloidal collectives break the water surface, they will be stably suspended on the water surface, supported by surface tension and surface tension-induced buoyancy force. Colloidal collectives are no longer affected by flowing fluid when turning off the optical field; thus, the analysis model for colloid collectives when they enter the water is given by (*59*)

$$F_{B}+F_{S}<F_{G}$$ (4)

The water entry of the colloidal collectives can be achieved by reducing the surface tension and buoyancy. Surface tension is mainly determined by the contact angle and length of the colloidal collectives, while buoyancy is determined by the contact area (see note S10 and fig. S18 for more details). The contribution of surface tension is more notable for lighter masses (<1 g) than buoyancy, but buoyancy becomes more important for heavier (>1 g) objects as the contact area grows faster than the contact length with increasing size. Because of the light mass of the colloidal collectives (≈0.006 g), the surface tension mainly contributes to the net upward force of the colloidal collectives. To achieve their submersion into the water without dispersion, surface tension must be reduced while keeping the magnetic field on, which can be realized by adjusting the colloidal collectives’ contact posture with the water surface. Here, increasing the pitch angle of the rotating magnetic field then drives the colloidal collective to flip on the water surface. Therefore, the net contact length of the flipped colloidal collective is reduced. When the colloidal collective is actuated to a 90° state, the net contact length with the water surface changes from 3 to 0.5 mm, markedly reducing the surface tension (fig. S18). As shown in Fig. 4B, in the initial state (*t* = 0 s), the rotating colloidal collective suspends on the water surface; when the pitch angle of the magnetic field increases to 20° (*t* = 6 s), the colloidal collective starts to sink under the gravity (movie S8). The external rotating magnetic field is applied all the time, preventing the colloidal collective from dispersing during its sinking. Moreover, the colloidal collectives can be controlled to sink at the desired time by adjusting the magnetic field instantly.

### Adaptive locomotion in the 3D air-aquatic environment

**Fig. 5.**
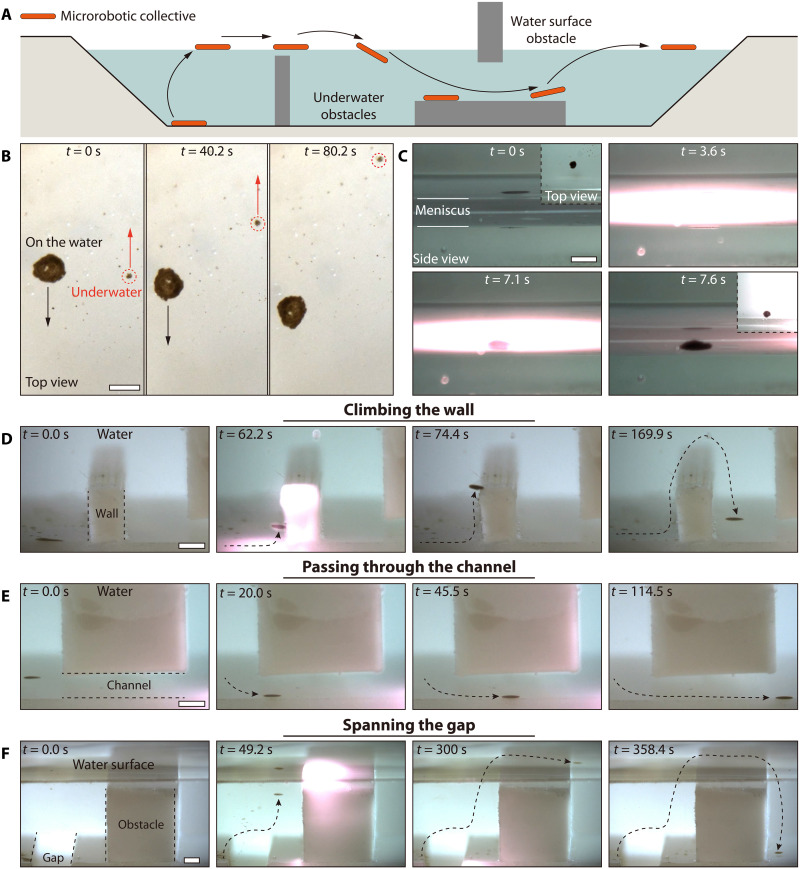
Adaptive locomotion of the microrobot collective. (**A**) Illustration of the microrobot collective locomotion underwater and at the air-water interface among 3D obstacles. The microrobot collectives can move underwater, maneuver on the water surface, dive into water, and make transitions between the water surface and the underwater environment. (**B**) Microrobot collective moves on the water surface under the magnetic field (*f*: 50 Hz, *B*_m_: 9 mT, θ: 10°). (**C**) Microrobot collective climbs up the water meniscus under the optical field. (**D**) A collective crosses an obstacle with a height of 10 mm. (**E**) Microrobot collective passes through a channel with a diameter of 2.5 mm (*f*: 50 Hz, *B*_m_: 9 mT, θ: 10°). (**F**) Microrobot collective crosses a gap with a width of 10 mm and climbs the high obstacle along the water-air interface. [(A) to (F)] Scale bars, 3 mm.

Conventional microrobot collectives rely on physical boundaries to introduce spatially asymmetrical interactions for locomotion (*13*, *20*, *21*, *23*, *25*, *26*, *30*), and typically only move underwater (*60*) or on the water’s surface (*61*–*63*). However, this dependence on physical boundaries limits the maneuverability and application scenarios of microrobot collectives. For example, it is hard to navigate microrobot collectives to a target location inside an environment without wall-bounded support. Although ultrasound can produce virtual walls, the ultrasound field is easily affected by the environment, and the whole device is not very scalable (*31*, *64*). Swarms formed by helically structured particles cannot keep the whole from dispersing in 3D motion (*48*–*50*). In addition, the lack of 3D motion capabilities renders the microrobot collectives incapable of performing simultaneous underwater and above-water operational tasks. Developing microrobot collectives that can adapt to a 3D bimodal air-aquatic environment is a great challenge. Here, colloidal collectives can be used as microrobot swarms with the capabilities of locomoting in 3D space and adapting to a bimodal air-aquatic environment (Fig. 5A). Microrobot collectives reaching above the water surface are not only stably suspended on the water surface but also able to perform controlled motions on the water surface. Magnetic and optical fields can drive the movement of microrobot collectives on the water surface, but the principles of magnetic and optical fields differ. As these microrobot collectives move on the water surface, they experience a combination of forces, including the form drag force *F_d_* = ρ*U*^2^*A*, the buoyancy force *F*_B_ = ρ*ghA* (primarily vertical but may contain a horizontal component), the added inertia force $F_{a}=\rho V\frac{dU}{dt}$, the viscosity drag force $F_{v}=\mu U\frac{A}{w}$, the surface tension force $F_{S}=\gamma \frac{A}{w}$ (also has a horizontal component), and the Marangoni force *F_m_* = ∇ γ*A*. The combination of these forces can be expressed as follows (*59*, *65*):

$$|F|\;∼\rho U^{2}A+\rho ghA+\rho V\frac{dU}{dt}\;+\mu U\frac{A}{w}\;+\gamma \frac{A}{w}\;-\nabla \gamma A$$ (5)

where ρ is the density of water, *g* is the gravitational constant, μ is the viscosity of water, *U* is the speed of the body, *V* is the characteristic volume of the body in consideration, *A* is the characteristic area of the body, *w* is the characteristic width of the body, *h* is the depth of the body from the original water surface, and γ is the surface tension coefficient. The magnetic field–driven method can determine the attitude of the microrobot collectives, which in turn changes the magnitude and direction of the surface tension and, thus, induces the collectives to move (see note S11 and fig. S19 for more details). As shown in Fig. 5B, when the pitch angle under the rotating magnetic field is 10°, the microrobot collective undergoes directional motion on the water surface in the opposite direction to that on the substrate (see movie S9 for more details). The field-induced rotation of the tilted collectives will generate a flow that induces the translation once rectified by the presence of the external boundary. Although the interfaces in both cases bind the motion of the colloidal collectives, the local flow induced by the stick boundary condition characteristic of the solid wall differs from the air-liquid interface, leading to an opposite direction of the collectives. As described in the previous section, when the pitch angle of the rotating magnetic field increases to 20°, the microrobot collective sinks to the bottom of the water due to a sharp decrease in surface tension. For the light-driven method, the Marangoni effect can be induced in microrobot collectives, which changes the magnitude and direction of the Marangoni force and thus causes the collectives to move (see note S12 and fig. S20 for more details). As shown in Fig. 5C, the microrobot collectives can climb the water meniscus with a height of 5 mm driven by the optical field.

Traditional microrobot collectives are known as “surface walkers” and are limited in their ability to cross obstacles larger than their own size. However, our proposed microrobot collective can adapt to complex terrain. We tested the locomotion capability of the collectives in different complex environments (movie S10). When a collective (thickness: 0.5 mm, length: 3 mm) encounters an obstacle with a height of 10 mm, it can easily use convective flow to move beyond the wall. Figure 5D indicates the colloidal collectives cross the barrier within 170 s. Owing to the dual response characteristics of the collectives, they can be driven by magnetic fields even when they encounter areas that are difficult to irradiate by optical fields. As shown in Fig. 5E, it is difficult for light to reach the microrobot collective directly due to the presence of obstacles above. In this case, the magnetic field can be adjusted to actuate the microrobot collective. By increasing the pitch angle of the magnetic field from 0° to 10°, a microrobot collective can pass smoothly through a narrow channel with a height of 2.5 mm. Even with a wide gap and obstacles close to the water surface, microrobot collective can use their 3D motion ability to overcome quickly. As shown in Fig. 5F, the width of the gap is 10 mm, the depth is 5 mm, and the height of the barrier is 20 mm, which is already close to the water’s surface. The conventional microrobot collectives need to rely on the substrate or interface for movement, and it is difficult to get out when they fall into the bottomless gap. There is also no way to face an exceptionally high obstacle. Our presented microrobot collectives can use convection to cross the gap, reaching the water surface and bypassing high barriers.

## DISCUSSION

In this study, inspired by the migration mechanism of plankton, we use a bimodal actuation strategy (combining magnetic and optical fields) in the nonliving active matter systems to propel the colloidal collectives to move in 3D space without boundaries. The combining fields enable the formation and controlled 3D locomotion of active colloidal collectives in an aquatic environment. With the programmed input of magnetic and optical fields, colloidal collectives can perform managed transitions at the water-air interface and respond to magnetic or optical fields for controlled motions on the water surface. Other collective systems also make it possible to realize the 3D drifting of particle collectives without dispersion. They should have the following characteristics: The particles need to have both a photothermal effect and a magnetic response; the density of the particles should be as small as possible, i.e., the upward lifting force generated by the convection induced by the photothermal effect should be able to overcome its gravity; the interaction force between particles within the particle collectives should be large enough to ensure that the collectives stay intact during the 3D motion. Thus, this bimodal actuation strategy can be used to drive other particle collectives to achieve their 3D motion without dispersion.

The presented colloidal collectives may provide a powerful tool for exploring the physics of self-assembly and a practical method for synthesizing functional materials. The complex form and functions of living systems are underpinned by molecular self-assembly, and a primary goal of materials science is to create synthetic materials based on particle assembly. The proposed 3D manipulation of self-assembled colloidal collectives can further build more sophisticated assembled structures using colloidal collectives as building blocks of materials. Under the external magnetic field, it is possible to create colloidal structures that do not represent the most thermodynamically stable arrangements. Guidance through spatial confinement or by interfaces provides the means for attaining unusual geometries and patterns. As shown in fig. S21A, two colloidal collectives are suspended by stacking them vertically between two vertical walls and keeping them spaced apart, using the ability of the colloidal collectives to move in three dimensions. Stacking multiple colloidal collectives vertically and varying the structure’s height is also possible by controlling the optical field’s intensity (fig. S21B). Suppose these can be locked into place by forming strong interactions. In that case, it becomes possible to achieve molecular analogs of kinetically stabilized structures, including very open structures that have been elusive for colloidal assemblies. The next step is to investigate how to lock the collectives and create complex materials or designs.

Dual-responsive colloidal collectives can serve as microrobot collectives with excellent environmental adaptability for controlled 3D motions in biofluids with high viscosity and high ionic concentration and are not constrained by the complexity of the environment (fig. S22). Thus, our approach offers a viable alternative to boundary-based propulsion over complex terrain in different fluids. Magnetic and optical actuation methods currently have different pros and cons, as the most widely used in microrobotics. They can enable long-range, fast, and precise actuation of microrobots in diverse environments. Magnetic actuation has unique potential for medical applications of microrobots inside nontransparent tissues at high penetration depths, while the optical one is suitable for more biotechnology, lab/organ-on-a-chip, and desktop manufacturing types of applications with much less surface penetration depth requirements or with transparent environments. Combining both methods in magneto-optic material actuation applications for colloidal assembly, magnetically programmable optical surfaces, or environmental remediation could have a strong potential of combining the pros of both approaches.

Moreover, another challenge in microrobotic collectives is the selective control of multiple untethered collectives. Unlike traditional robots, microrobots are challenging to equip with actuators and onboard sensors for motion and control. Therefore, independent management of multiple individual microrobots is already difficult because all microrobots receive the same control inputs in the single external magnetic field, let alone independent control of microrobotic collectives. It’s worth noting that the magnetic field is global, and the operating area of the optical field is adjustable and selective. The magnetic field excites the self-assembly of colloids and maintains the self-assembled microrobotic collectives without disassembly, while the optical field drives selected microrobotic collectives to perform different tasks. Therefore, the bimodal actuation strategy that combines the magnetic field with the optical field is a feasible solution to achieve selective control of magnetic microrobotic collectives.

## MATERIALS AND METHODS

### Materials and setup for experiments

The colloidal particles were prepared by sonication of hydrocarbon oil-based ferrofluid droplets. The hydrocarbon oil-based ferrofluids in our work were purchased from Taobao, China, with a dynamic viscosity of 50 cP, a saturation magnetization of 43 mT, and a density of 1.29 g/ml (the information detail of ferrofluid is shown in table S1). One milligram of ferrofluid droplets was added to 10 ml of water and then sonicated in an ultrasonic device (KQ3200DB: 100 W, 40 kHz) for 1 hour to obtain colloidal particles with a particle size of below 1 μm. The magnetic actuation setup comprises three orthogonal pairs of custom-made electromagnets, and it has an internal chamber size of 50 mm by 50 mm by 50 mm (fig. S2). Software control signals specify the input currents driving the electromagnets through a custom electronic board. The intensity of the magnetic field is adjustable from the field off to a maximum of 9 mT. A transparent tank of size 20 mm by 20 mm by 10 mm made using acrylic plates filled with deionized water solution was used to experimentally observe the behavior of colloidal collectives. The light source used in our work was 808-nm NIR light from Hi-Tech Optoelectronics (LOS-BLD-0808-15 W-C/P). The power of the optical field is adjustable from 0 to 15 W. The 3D printer (Pro2, RAISE3D) was used to construct a variety of terrain used in the experiment, such as gaps, walls, and channels.

### Applied magnetic field

The rotating magnetic field is schematically depicted in fig. S3A, and the magnetic field vector **B** is defined as

$$B(t)=B_{m}[\cos(2\pi ft)e_{x}-\sin(2\pi ft)e_{y}]$$ (6)

where *B*_m_ is the amplitude of the rotating magnetic field, *f* is the rotating frequency, *t* is time, and **e***_x_* and **e***_y_* are the unit vectors along the *x* axis and *y* axis, respectively. The pitch angle θ defines the magnetic field tilt relative to the *x-y* plane. The profiles of the rotating magnetic field **B** with a field strength *B*_m_ of 1 mT, a frequency *f* of 1 Hz, and pitch angles of 0°, 45°, and 90° are plotted in fig. S3B, respectively. The 3D, top, and side views illustrate the dynamic magnetic field superposition pattern.

### Characterization

We used a scanning electron microscope (SEM) (Quanta 400F, FEI Company) to observe the morphology of ferrofluid colloids. For the SEM experiment, the ferrofluid colloid dispersed in the aqueous phase needs to be dried: First, take 5 μl of the colloidal dispersion, then disperse it on the silicon wafer substrate, and lastly put it in a fume hood to stand for 12 hours. The dynamic light scattering sensor (NANOPHOX, Sympatec) was used to perform a size analysis of the ferrofluid colloids.

### Video acquisition and analysis

The camera (MER-503-36U3C, Daheng Imaging) was used to observe and record the motion behavior of colloidal collectives. The infrared camera (FLIR ONE Gen 3, FLIR SYSTEMS) was used to observe the temperature changes induced by the colloidal particles and colloidal collectives. The translational velocity of colloidal collectives was analyzed by video analysis in ImageJ.

### Simulations

On the basis of Eqs. 1 and 6, we simulated the motion behavior of colloidal particles using our own interface programmed with LabVIEW software to study the formation process of colloidal collectives under the rotating magnetic field. The colloid diameter was set at 500 nm, the colloid density was set at 1290 g/m^3^, and the magnetic susceptibility was set at 0.28. The strength of the magnetic field was set at 9 mT and the frequency was varied from 1 to 100 Hz. We chose to solve numerically in convection of the colloidal collectives using COMSOL Multiphysics, a commercial software based on finite element calculations. Because of the axial symmetry of the investigated system, a 2D model was considered. The Pardiso solver was used on a free triangular mesh. The heat transfer and laminar flow modules were used to solve for transient values of coupled fields. The physical parameters of water were taken from the COMSOL library, and their temperature dependence was considered. No slip boundaries are considered and set as thermal insulation. The diameter of the colloidal collective is set at 4 and 0.5 mm from the lower boundary of the flow field. The length and height of the tank are set to 30 and 10 mm, respectively. The parameters of water in the simulation are summarized in table S4.

## Supplementary Materials

### This PDF file includes:

Notes S1 to S14 Figs. S1 to S22 Tables S1 to S4 Legends for movies S1 to S10
