# Supplementary material for: Bioinspired self-assembled colloidal collectives drifting in three dimensions underwater
Source: Sci Adv. 2023 Nov 10;9(45):eadj4201. doi: 10.1126/sciadv.adj4201 (PMC10637755; doi:10.1126/sciadv.adj4201)
Supplement: Supplementary file 1 — Notes S1 to S14 Figs. S1 to S22 Tables S1 to S4 Legends for movies S1 to S10 [file sciadv.adj4201_sm.pdf]

Science Advances
AAAS

Supplementary Materials for

Bioinspired self-assembled colloidal collectives drifting in three dimensions underwater

Mengmeng Sun *et al.*

Corresponding author: Li Zhang, lizhang@cuhk.edu.hk; Metin Sitti, sitti@is.mpg.de

Sci. Adv. **9**, eadj4201 (2023)
DOI: 10.1126/sciadv.adj4201

The PDF file includes:

Notes S1 to S14
Figs. S1 to S22
Tables S1 to S4
Legends for movies S1 to S10

Other Supplementary Material for this manuscript includes the following:

Movies S1 to S10

Supplementary Notes

Supplementary Note 1. Preparation and characterization of ferrofluid colloid particles

Supplementary Figure 1a displays photo images of ferrofluid droplets that were transformed into colloidal particles using ultrasonic dispersion.The information detail of ferrofluid is shown in **Supplementary Table 1**. For the scanning electron microscope (SEM) experiment, the ferrofluid colloid dispersed in the aqueous phase needs to be dried. To do this, take 5 μL of the colloidal dispersion and distribute it onto a silicon wafer substrate. Next, place the substrate in a fume hood and let it stand for 12 hours. The SEM image of ferrofluid colloid particles shows a film on the outside (**Supplementary Fig. 1b**). This film forms because the ferrofluid colloidal particles are nanoscale oil droplets that create a film during drying. As shown in **Supplementary Fig. 1c**, the dynamic light scattering (DLS) results of the ferrofluid colloids solution at 0.1 mg/mL indicate that the size of the ferrofluid colloids was under 1 μm.

**Appearance:** Black fluid	**Viscosity @27°C:** <5 mPa·s
**Iron Oxide (magnetite):** 9% (by volume)	**Density @25°C:** 1.29 g/cc
**Oleic Acid Dispersant:** 18% (by volume)	**Pour Point:** -94 °C
**Distillates (Petroleum) Hydrotreated Light:** 73% (by volume)	**Flash Point:** 89 °C
**Nominal Particle Diameter:** 10 nm	**Initial Magnetic Susceptibility:** 0.28
**Saturation Magnetization:** 44 mT	**Water Solubility:** Insoluble

Supplementary Table 1 | Composition, physical and chemical properties of ferrofluid.

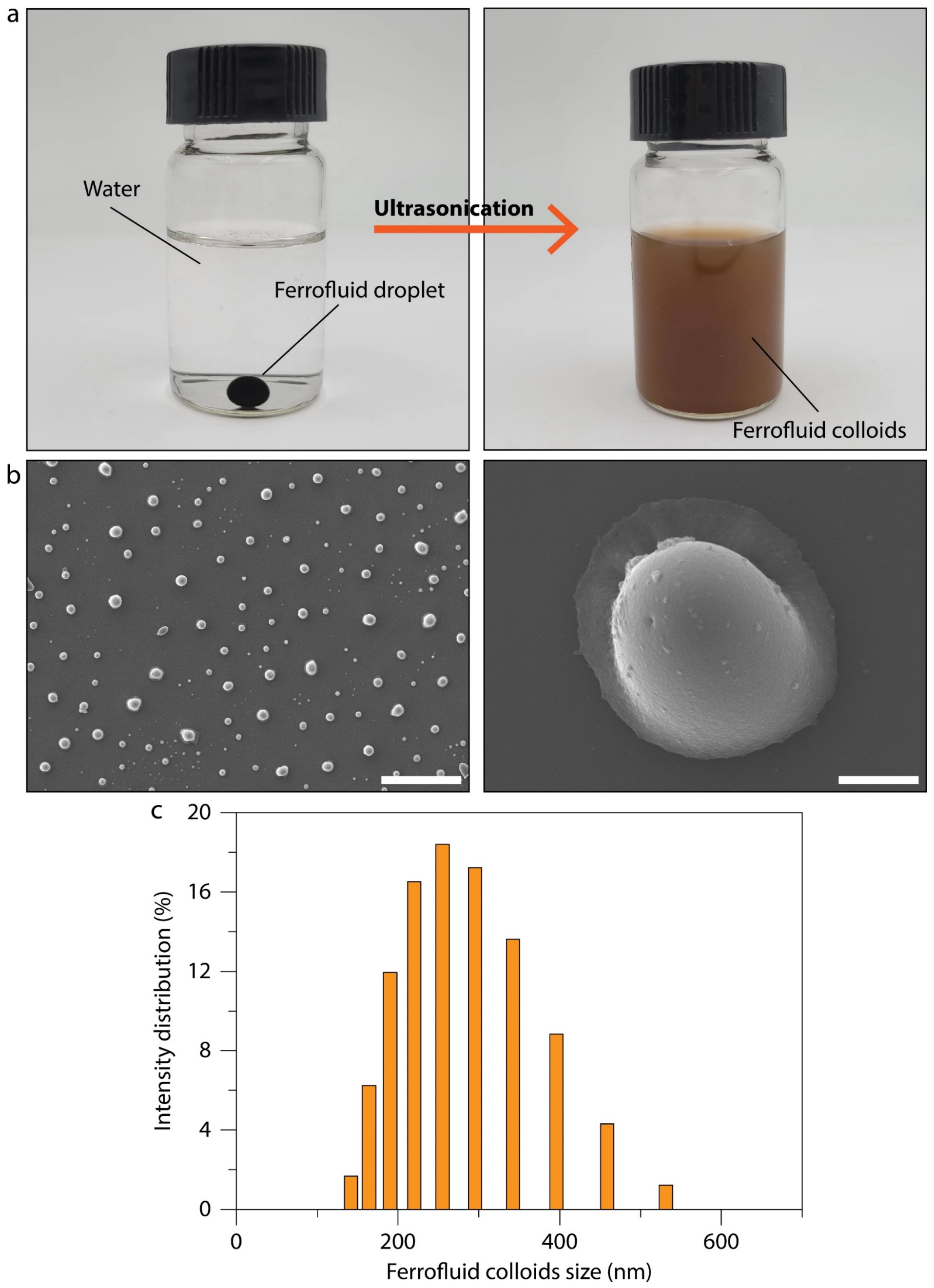

Supplementary Figure 1 | Preparation and characterization of ferrofluid colloid particles. a, Photo images of ferrofluid droplets dispersed in the aqueous phase before and after sonication for one hour. **b,** SEM images at different magnifications confirm the existence of thin films on the periphery of the ferrofluid colloids after drying. Scale bars, 1 μm. **c,** DLS result of ferrofluid colloids solution at 0.1 mg/mL in water.

Supplementary Note 2. Experimental setup

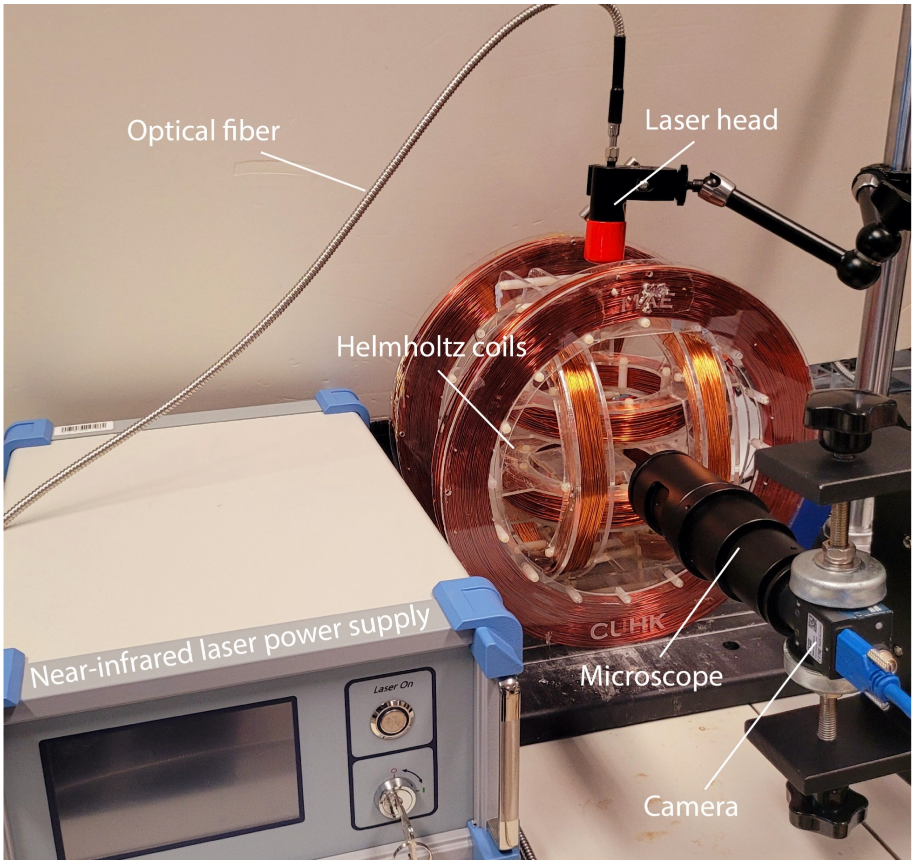

Supplementary Figure 2 | Photo of three orthogonal pairs of custom-made electromagnets setup combined with near-infrared laser used to actuate the colloidal collectives. The power density is shown in Supplementary Table 2.

**Power:** W	**1**	**2**	**3**	**4**	**5**
**Indensity:** W/mm^2	**0.0205**	**0.041**	**0.0615**	**0.082**	**0.1025**

Supplementary Table 2 | Laser power density.

Supplementary Note 3. Applied magnetic field

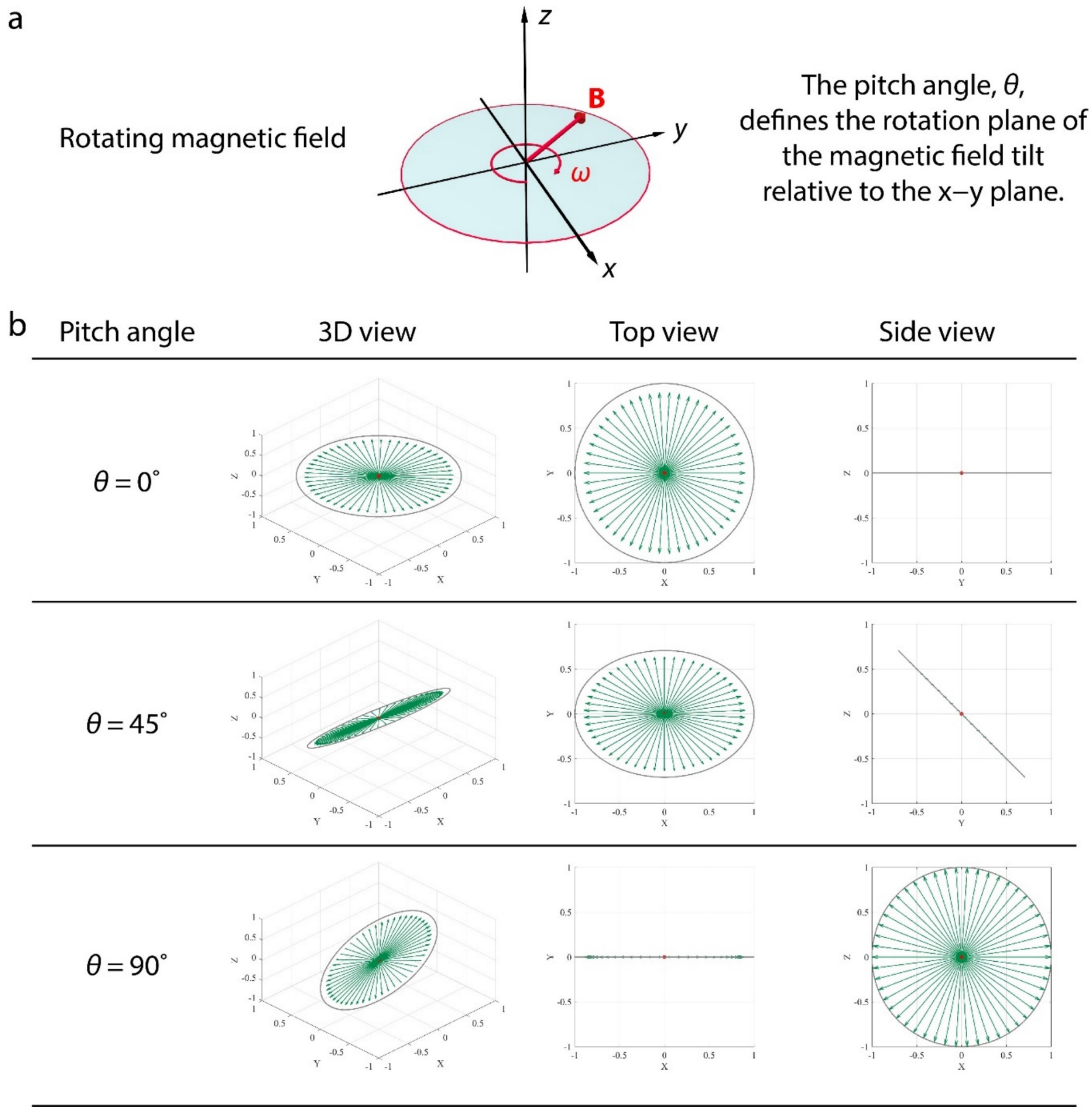

Supplementary Figure 3 | Applied magnetic field. a, Schematics showing the applied rotating magnetic field. **b,** Profile of the magnetic field when the pitch angle is 0°, 45°, and 90°, respectively.

Supplementary Note 4. Theoretical model for the generation of colloidal collectives

When energized by a rotating magnetic field, dispersed ferrofluid colloids interact with each other and assemble into colloidal collectives. In this study, we analyzed the forces experienced by a colloidal particle. The colloidal particles were assumed to have the same radius (a), density (ρ), volume (V), and effective magnetic susceptibility (χ). Under a static external magnetic field (**B**), a paramagnetic colloid acquires an induced moment (***m***)

$$\boldsymbol{m} = V\chi\mathbf{B}/\mu_0 \tag{2}$$

Where $V = 4\pi a^3/3$ represents the particle volume, and $\mu_0 = 4\pi\times10^{-7}$ H/m is the vacuum permeability. At sufficiently high frequencies, the rotating field induces attractive dipolar interactions that are isotropic when time-averaged. The dipolar interaction between two equal dipoles $\boldsymbol{m}_{i,j}$ at a distance $\boldsymbol{r}_{ij} = \boldsymbol{r}_i - \boldsymbol{r}_j$ is given by

$$U_m = -\mu_0/4\pi\{[3(\boldsymbol{m}_i \cdot \boldsymbol{r}_{ij})(\boldsymbol{m}_j \cdot \boldsymbol{r}_{ij})/r^5] - (\boldsymbol{m}_i \cdot \boldsymbol{m}_j)/r^3\} \tag{3}$$

Which becomes maximally attractive (repulsive) for particles with magnetic moments parallel (normal) to $\boldsymbol{r}_{ij}$. Performing a time average of the potential gives an effective attractive interaction in the *x*-*y* plane

$$\langle U_m \rangle = -\mu_0 m^2/[8\pi(x+y)^3] \tag{4}$$

Thus, a random dispersion of colloidal particles that would otherwise perform simple Brownian motion, are forced to assemble into a compact collective. And the magnetic dipole-dipole interaction force $\boldsymbol{F}_i^m$ can be expressed as

$$F_i^m = \sum_{i \neq j} \frac{3\mu_0}{4\pi r^4} \{5(\boldsymbol{m}_i \cdot \boldsymbol{n})(\boldsymbol{m}_j \cdot \boldsymbol{n})\boldsymbol{n} - (\boldsymbol{m}_j \cdot \boldsymbol{n})\boldsymbol{m}_i$$

$$-(\boldsymbol{m}_i \cdot \boldsymbol{n})\boldsymbol{m}_j - (\boldsymbol{m}_j \cdot \boldsymbol{m}_i)\boldsymbol{n}\} \quad (5)$$

Where r is the particle distance, and $\boldsymbol{n}$ is the normal vector pointing from particle j to i. i and j indicates the i-th and j-th particles. In addition, the hydrodynamic drag force $\boldsymbol{F}_i^d$, and gravitational force $\boldsymbol{F}_i^g$ can be expressed as

$$\boldsymbol{F}_i^d = 6\pi\eta a \boldsymbol{v_i} \quad (6)$$

$$\boldsymbol{F}_i^g = -\frac{4}{3}\pi a^3(\rho - \rho_f)g\hat{z} \quad (7)$$

Where $\boldsymbol{v}_{\mathrm{i}}$ is the velocity of particle i, η is the dynamic viscosity of the fluid, g is the amplitude of the gravity field, ρ_f is the density of the fluid and $\hat{z}$ is a normal vector pointing $+z$ direction.

The movement of particles through the fluid will influence the corresponding flow field, and consequently, the movements of the particle i is affected. In this study, we neglect inertia of the liquid and assume Stokes flow. Hydrodynamic interactions among the particles are analyzed by considering the stokeslet of spherical particles. The hydrodynamic force $\boldsymbol{F}_i^h$ exerted on the liquid at the position of i-th particle by particle j is equal in magnitude but opposite in direction to the hydrodynamic drag force $\boldsymbol{F}_j^d$ experienced by particle j, that is, $\boldsymbol{F}_i^h = -\boldsymbol{F}_j^d$. In view of the linear Stokes equation, it is natural to assume that the velocity perturbations caused by the different colloidal particles can be superimposed, i.e., the total velocity perturbation of the flow field at position $\boldsymbol{r}_i$ caused by (N-1) particles equals

$$\boldsymbol{u_i} = \sum_{j=1, j \neq i}^{N} \boldsymbol{O}(\boldsymbol{r}_{ij})\ \boldsymbol{F}_i^h \quad (8)$$

Where the $\boldsymbol{O}(\boldsymbol{r}_{ij})$ is the Oseen-Burgers tensor.

The repulsive force is introduced in order to avoid overlap between colloid-colloid and colloid-wall, and the force is present only when the distance is less than the natural length.

$$\boldsymbol{F}_m^{rep} = \sum_{r<r_0} k(r - r_0)\boldsymbol{n} \tag{9}$$

Where k is the spring constant, and r_0 is the natural length.

Ignoring the negligible inertia of the particles, from Eq. (5) to (9) the governing equation for the velocity of i-th colloidal particle is given by

$$\boldsymbol{F}_i^d = \boldsymbol{F}_i^m + \boldsymbol{F}_i^g + \boldsymbol{F}_i^h + \boldsymbol{F}_m^{rep} \tag{10}$$

$$\boldsymbol{v_i} = \frac{\boldsymbol{F}_i^m + \boldsymbol{F}_i^g + \boldsymbol{F}_m^{rep}}{6\pi\eta a} + \boldsymbol{u_i} \tag{11}$$

From Eq. (11), the velocities of the particles are influenced by the gravity, surrounding flow, and time-varying field-induced magnetic interactions.

The velocity terms of the droplets are integrated and stripped out to solve the dynamics of the coupled fluid field in real-time, thereby indicting the droplet motion. A series of collective behaviors of multiple droplets can then be simulated. The collective behavior between 78 colloidal particles was simulated based on Eq. (11) and is shown in **Supplementary Fig. 4 (**the parameters setting is shown in **Supplementary Table 2)**. The field strength B_m, and pitch angle of the applied rotating magnetic field are 5 mT and 90°, respectively. Upon energized, the colloidal particles interact with each other. The collective behavior of colloidal particles evolves significantly with the input magnetic field frequency from 1 to 20 Hz. At the low frequency (less than 3 Hz), the colloids assemble to form chain-like structures, and multiple chain-like structures perform synchronous rotation to form

irregular colloidal clusters. The dynamics of the chain-like structures on the *x-y* plane, which is governed by the Mason number (Ma: ratio of viscous to magnetic forces), which can be defined as

$$\mathrm{Ma} = \frac{144\omega^2}{\mu 0 \mu_s M^2} \tag{12}$$

Where η, ω, μ_0, μ_s, and M are, respectively, the solvent viscosity, the angular frequency of the magnetic field, the vacuum magnetic permeability, the solvent magnetic permeability, and the magnetization of the colloidal particles. As the frequency increases, the clusters are finally broken into shorter chain clusters. The chain clusters become shorter with an increase in the frequency and those short clusters coagulate to form isotropic disk-like clusters on the plane of the rotating magnetic fields.

Particle radius a	Magnetic susceptibility χ	Particle density ρ	Vacuum permeability μ_0	Fluid density ρ_f	Fluid viscosity η	Gravitational acceleration g	Magnetic field strength B	Magnetic field frequency f	Interparticle collision diameter r_0
0.5 [μm]	0.28	1290 [kg/m^3]	$4\pi\times10^{-7}$ [H/m]	1000 [kg/m^3]	1.0×10^{-3} [Pas]	9.8 [m/s^2]	0-10 [mT]	0-50 [Hz]	1 [μm]

Supplementary Table 3 | Parameters used in simulation and analysis.

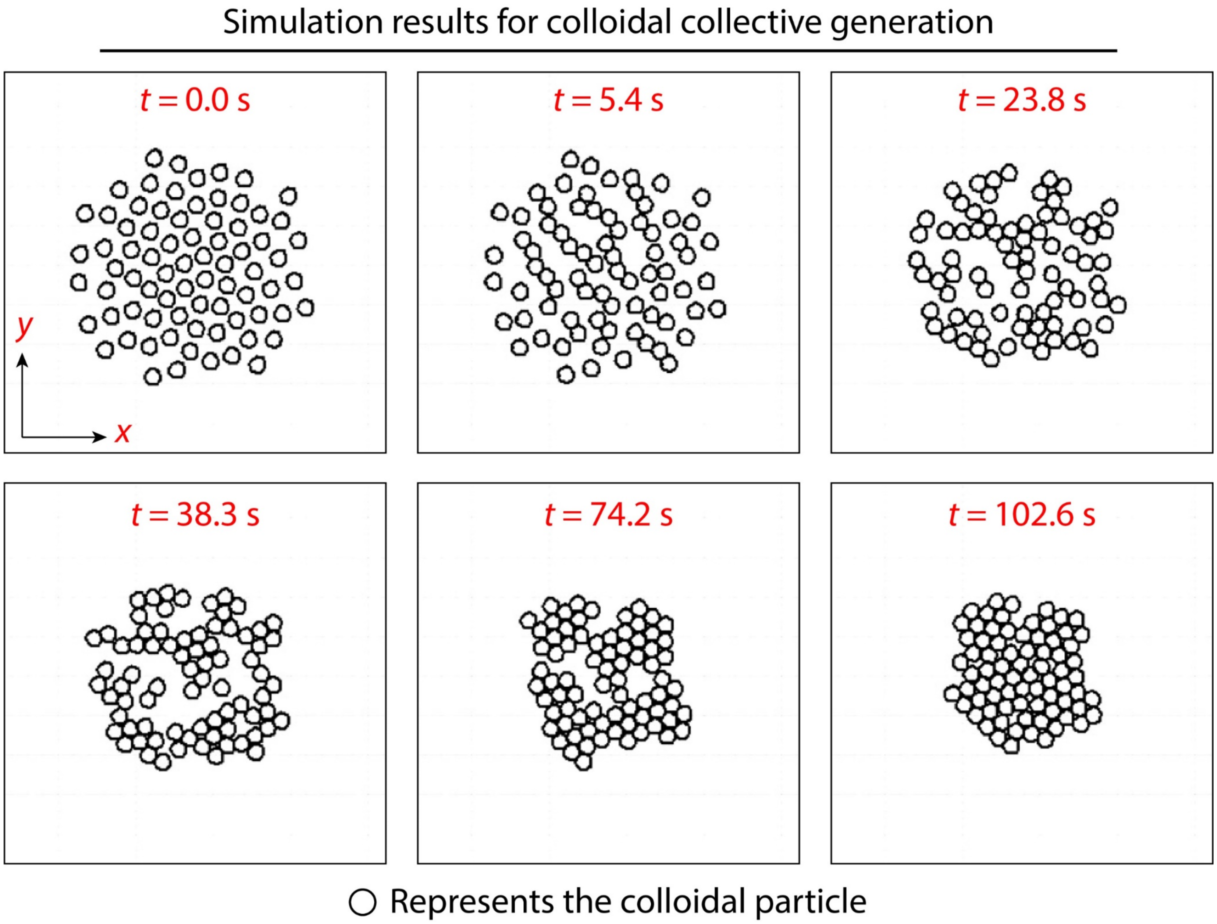

Supplementary Figure 4 | Simulation of a colloidal collective generation processes. Hollow circles represent colloidal particles.

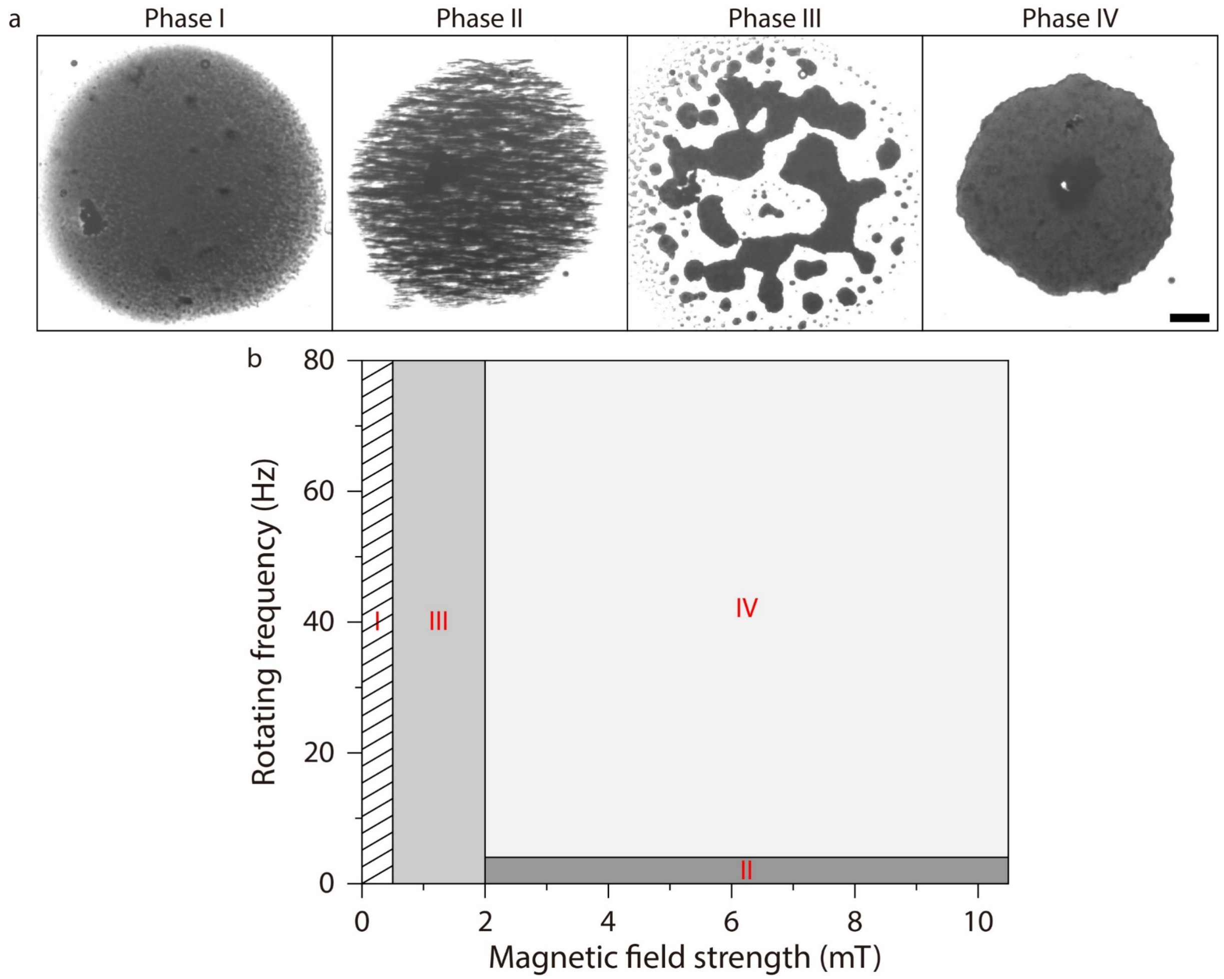

Supplementary Figure 5 | Self-assembled colloidal collectives. a, Different phases of the colloidal collectives. Scale bar, 100 µm. **b,** Phase diagram showing the colloidal structures generated in the magnetic fields with different combinations of the magnetic field strength B_m and frequency f. The corresponding phases of the colloidal structures are represented by I, II, III, and IV, respectively.

The study investigates the colloidal structures that arise from applying different combinations of magnetic field strength (B_m) and frequency (f). The experiments use the same colloidal particles throughout. **Supplementary Fig. 5a** shows that, in the absence of a magnetic field or under a low-intensity, high-frequency rotating magnetic field, the colloidal particles exhibit Brownian motion on the substrate. However, as the magnetic field strength increases, the colloidal particles form chain-

like clusters that occupy a slightly smaller overall coverage area (phase II). As the strength and frequency of the magnetic field increase further, the chain-like clusters merge, forming multiple vortex-like collective structures (phase III). Under the magnetic field conditions shown in phase IV, an entity can be created. In this region, most of the colloidal particles successfully aggregate. **Supplementary Fig. 5b** presents the phase diagram that illustrates the various self-assembled colloidal structures.

Supplementary Note 5. Gravity-resisting characteristics of the colloidal collectives

By varying the pitch angle of the rotating magnetic field, it is possible to keep the colloidal collectives intact during overturning motion. The tangential component of the dipole magnetic interaction force drives the overall overturning motion of the colloidal collectives. To simplify the flipping motion of colloidal collectives, we represent it as the flipping motion of chain-like structures (**Supplementary Fig. 6a**). As depicted in the diagram, the dispersed colloidal particles form a colloidal collective at time t_1 and then lie flat on the substrate. The angle θ between the rotating magnetic field and the horizontal plane is then increased to drive the entire colloidal collective to flip, flipping to a position where the angle with the horizontal plane is α at time t_2. The driving magnetic torque T_m of the N-particle chain, subjected to the applied magnetic field during the overturning process, can be expressed as

$$T_m = \frac{3\mu_0 m^2 (N-1)}{4\pi(2a)^3} \sin\left(2(\theta - \alpha)\right) \tag{13}$$

Where $\mu_0 = 4\pi\times10^{-7}$ H/m is the vacuum permeability, m is the induced moment of colloid, and a is the radius of colloidal particle.

During the flipping motion, the chain-like structures will suffer from the resistive viscous torque T_d. Unlike a chain that rotates on the x-y plane, whose rotation center is in the middle of the chain. And the rotating chain on the x-z vertical plane has its center of rotation at one end of the chain. Therefore, under the same angular frequency, the drag torque on the chain-like structure in the x-z vertical plane is twice that on the chain-like structure in the horizontal plane, which can be expressed as

$$T_d = \frac{16\pi a^3}{3} \frac{N^3}{\ln\left(\frac{N}{2}\right) + \frac{2.4}{N}} \eta\omega \tag{14}$$

Where η is the dynamic viscosity of the fluid, and ω is the angular velocity.

Moreover, the gravitational torque T_g exerted on the chain can be expressed as

$$T_g = \frac{4}{3}(\rho - \rho_f) g \pi a^4 \cos(\alpha) N^2 \tag{15}$$

Where ρ is the density of the particles, ρ_f is the density of the fluid, and g is the amplitude of the gravity field.

The dimensionless Mason number is used to characterize the dynamics of a rotating chain in the *x*-*y* plane. Here, the fragmentations of the chains are significantly influenced by the number of composing particles and the angular velocity of the chain. Gravity plays a role in the flipping motion of the colloidal collectives. Therefore, a modified Mason number M_{ma} including the gravitational torque is derived, which can be expressed as

$$M_{ma} = \frac{T_d + T_g}{T_m} \tag{16}$$

The modified Mason number M_{ma} is derived based on the situation when the driven magnetic torque T_m is counterbalanced by the viscous torque T_d and gravitational torque T_g. When the modified Mason number M_{ma} is smaller than unity, it indicates that a colloidal collective flips without fragmentation. From Eq. (16), when the angular velocity of the magnetic field ω is considerably low, the viscous torque T_d is non-significant, a colloidal collective flip until the driven magnetic torque T_m is counterbalanced by the gravitational torque T_g. This means the colloidal collectives can be very large at this point, i.e., many colloidal particles self-assembly. In contrast, when the angular velocity of the magnetic field ω is high, the colloidal collective will undergo fragmentation or if it grows beyond the corresponding number of composing particles N.

The experiments investigated the flip process of colloidal collectives along the *y* and *x* axes, respectively, as shown in **Supplementary Figs. 6b** and **6c**. The strength of

the externally applied rotating magnetic field was 9 mT. From 0 to 81 s, the colloidal particles began to assemble to form colloidal collectives, during which the frequency of the external magnetic field was 10 Hz, and the input pitch angle was 0°. After 81s, the frequency of the external magnetic field increases to 100 Hz, at which point the entire colloidal collective transforms into a colloidal crystal. The input pitch angle is then adjusted so that it grows by 10°. The colloidal collective then flips over, driven by the magnetic torque. At 32 s, the colloidal collective flip to an angle of 45° to the substrate. During this process, the magnetic torque is sufficient to overcome the viscous and gravitational torque so that the whole remains intact without violent fracture. Increasing the pitch angle, the colloidal crystal has flipped to a position perpendicular to the substrate at 117 s. At this time, the magnetic and viscous torque are balanced, and the gravitational torque is 0. Subsequently, the pitch angle is increased to 180°, and the gravitational and magnetic torque overcome the resistive drag torque. After 213 s, the colloidal crystal is entirely flipped 180°. The magnetic field is turned off, the colloidal collective starts to disperse, and the colloidal particles begin to move in Brownian motion. With the same driving principle, the colloidal collective can also be flipped 180° along the *x*-axis (**Supplementary Fig. 6c**).

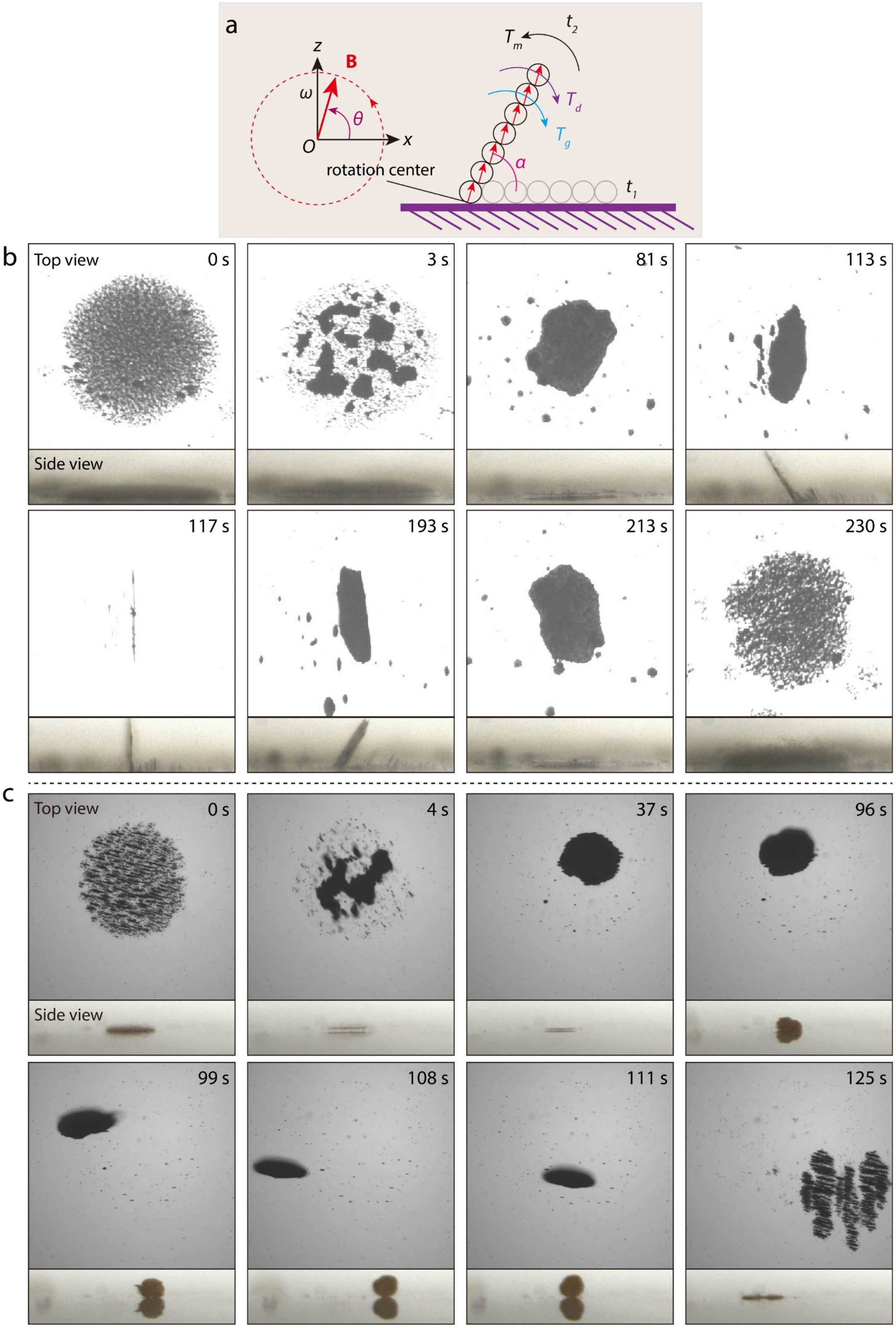

Supplementary Figure 6 | Flipping behavior of a colloidal collective under the rotating magnetic field. a, Schematics analyzing the rotation of a collective on the

substrate in the rotating magnetic field. Here, the colloidal crystal is simplified as a chain-like structure. The rotating angle of a crystal is represented by α. The angular velocity of the rotating magnetic field is represented by $\boldsymbol{\omega}(t)$. The phase lag angle between the magnetic field and the long axis of the pillar is represented by θ-α. The magnetic torque, gravitational torque, and viscous torque exerted on the pillar are represented by T_m, T_g , and T_d, respectively. **b,** Flipping motion of a colloidal collective along the *y*-axis over time. **c,** Flipping motion of a colloidal collective along the *x*-axis over time.

s

Supplementary Note 6. Photothermal effect of colloidal particles

The prepared ferrofluid colloidal particles can absorb near-infrared light and convert it into heat energy. Photothermal conversion efficiency is an important parameter to evaluate the photothermal properties of colloidal particles. To calculate the photothermal conversion efficiency, an energy balance on the system is required. The total energy balance for the system is

$$Q_{\mathrm{Mater}} + Q_0 - Q_{\mathrm{output}} = \sum m_i c_{p,i} \frac{dT}{dt} \tag{17}$$

Where Q_{Mater} is the heat generated by the ferrofluid colloidal particles under laser irradiation, Q_0 is the heat generated by solvent under laser irradiation, Q_{output} is the heat transferred from the system to the environment, m_i is the mass, and $c_{p,i}$ is the heat capacity

$$Q_{\mathrm{Mater}} = P(1 - 10^{-A_\lambda})\eta \tag{18}$$

$$\eta = \frac{Q_{\mathrm{Mater}}}{P(1 - 10^{-A_\lambda})} \tag{19}$$

Where P is the laser power, A_λ is the absorption intensity at the excitation wavelength of λ (nm), and η is the photothermal conversion efficiency.

$$Q_{\mathrm{output}} = hs(T - T_{\mathrm{surr}}) \tag{20}$$

Where h is the heat transfer coefficient, s is the surface area of the container, T is the solution temperature, and T_{surr} is the ambient surrounding temperature. In order to calculate hs, the cooling stage is studied. After removing the laser excitation, the heat generated by the system is stopped. Equation (17) becomes

$$\sum m_i c_{p,i} \frac{dT}{dt} = -Q_{\mathrm{output}} = -hs(T - T_{\mathrm{surr}}) \tag{21}$$

Rearranging Equation (21)

$$dt = -\frac{\sum m_i c_{p,i}}{hs} \frac{dT}{(T - T_{\mathrm{surr}})} \tag{22}$$

then integrating

$$t = -\frac{\sum m_i c_{p,i}}{hs} \ln{(T - T_{\mathrm{surr}})} + b \tag{23}$$

Let τ_0 be the time constant for heat transfer from the system

$$\tau_0 = \frac{\sum m_i c_{p,i}}{hs} \tag{24}$$

During solution cooling, the temperature decrease was monitored. τ_0 is calculated according to the temperature changes of the solution as a function of time. Thus, *hs* can be computed.

At the maximum steady-state temperature, the heat transfer between the system and the environment reaches equilibrium. The temperature is a constant.

$$\frac{dT}{dt} = 0 \tag{25}$$

Equation (17) gives the expression

$$Q_{\mathrm{Mater}} = Q_{\mathrm{output}} - Q_0 \tag{26}$$

Then the photothermal conversion efficiency can be expressed as

$$\eta = \frac{hs(T - T_{\mathrm{surr}}) - Q_0}{P(1 - 10^{-A_\lambda})} \tag{27}$$

In order to further study the photothermal properties of ferrofluid colloidal particles, we studied the temperature change of colloidal particles with time under the same power density and the maximum temperature difference of colloidal particles under different power densities, respectively. All experiments were carried out in a tank of 20 mm × 20 mm × 10 mm and the tank was filled with an aqueous solution, the concentration of colloidal particles was 0.5 mg/mL, the volume was 10 μL, and a

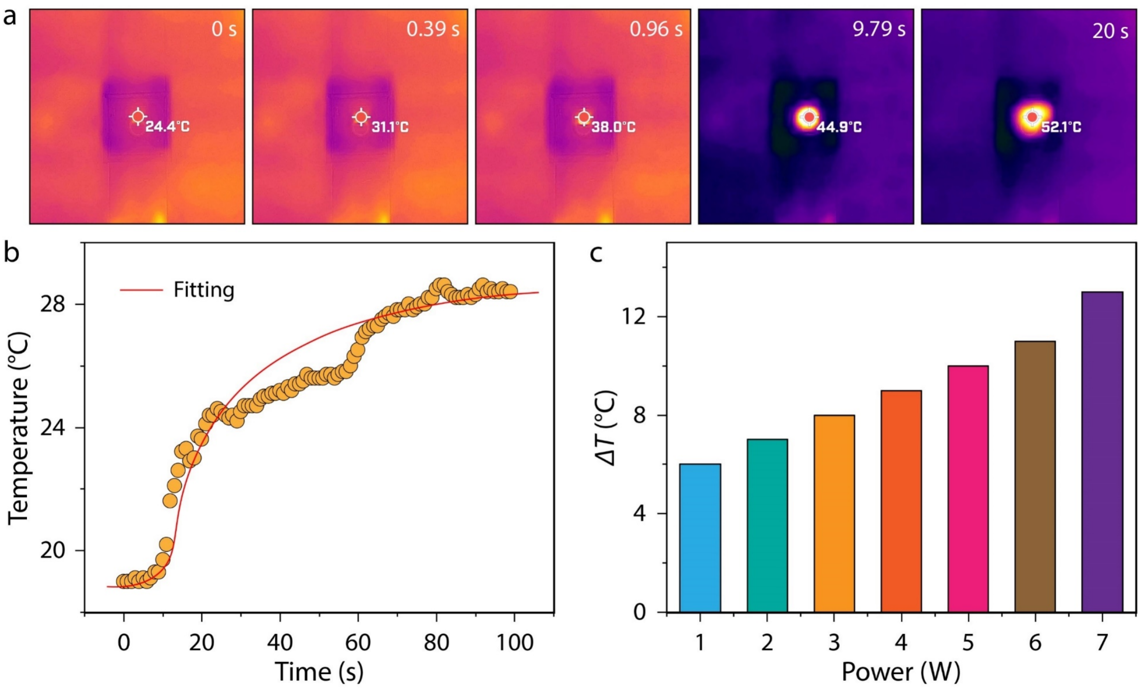

Supplementary Figure 7 | Photothermal effect of colloidal particles under the 808 nm laser irradiation. a, Thermal image acquired by the IR camera on the sample at the intermediate power (0.05 W mm^{-2}) whin 20 s of irradiation. **b,** Temperature increases of colloidal suspensions with time (laser wavelength: 808 nm, laser power density: 0.05 W mm^{2}). **c,** Irradiated by a laser with different power densities (concentration: 0.1 mg/mL, laser wavelength: 808 nm).

cylindrical permanent magnet with a diameter of 5 mm was used under the tank to enrich the particles. As shown in the **Supplementary Fig. 7a**, under the continuous irradiation of near-infrared laser, the thermal image of colloidal particles obtained by the infrared thermal imager (laser wavelength: 808 nm, laser power density: 1 W/cm2). Then, we recorded the temperature change of the aqueous solution in the tank with time, and the results showed that the temperature of the entire tank first increased and then remained stable under the irradiation of the near-infrared laser (**Supplementary Fig. 7b**). We also recorded the temperature change over time of colloidal particle suspensions under successively different power densities under

different concentrations of 808 nm laser irradiation, until the suspensions reached steady-state temperature. The results show that the temperature change of the colloidal particles increases with increasing power (**Supplementary Fig. 7c**).

Temperature and concentration affect the density of the flowing fluid resulting in a convective flow. When the laser illuminates a colloidal particles solution, the colloidal nanoparticles exposed to the resonant light will likely release thermal energy by photothermal conversion, giving rise to a local temperature gradient. This temperature gradient then produces a convective flow. Owing to the convective flow, the colloidal nanoparticles in the liquid experience a hydrodynamic force, moving along the streamline of the liquid. Compared with the hydrodynamic force, the optical gradient force is relatively small enough to be negligible. As shown in **Supplementary Fig. 8**, the dispersed colloidal particles deposited on a substrate are irradiated with a near-infrared laser. The colloidal particles induce a convective flow that drives the colloidal particles upwards (the surrounding liquid environment is water).

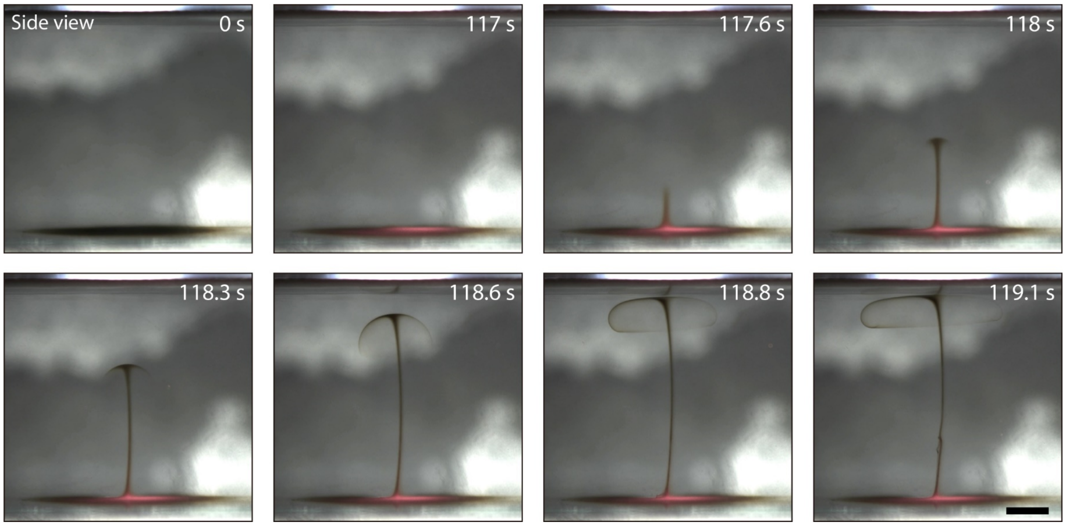

Supplementary Figure 8 | Convection of colloidal particles under the NIR laser beam. Fluid convection around the deposited colloidal particles in water under the irradiation of a near infrared laser. Scale bar, 100 µm.

Supplementary Note 7. The simulations and experiments of convective flow induced by the colloidal collectives

Similarly, there was also convective flow around the colloidal collectives due to the temperature difference between colloidal collectives and the surrounding liquid environment. The thermo-induced fluid motion expected around a colloidal collective under illumination originates from light absorption. This effect generates a temperature distribution $T(\mathbf{r},t)$ inside the collective governed by the heat diffusion equation:

$$\rho_c C_c \frac{\partial}{\partial t} T(\mathbf{r},t) - \kappa_c \nabla^2 T(\mathbf{r},t) = q(\mathbf{r}) \tag{28}$$

Where k_c is the thermal conductivity of gold, ρ_c its density, C_c its specific heat capacity at constant pressure, and $q(\mathbf{r})$ is heat power density.

Note that the temperature profile inside the collectives is usually very uniform despite the non-uniformity of the heat source $q(\mathbf{r})$. This is due to much higher thermal conductivity of colloidal collectives compared to that of water. Outside the structure, no heat is generated since the light absorption of water is negligible ($q(\mathbf{r}) = 0$), but some convection may occur. Hence, the temperature distribution in the surrounding water is governed by the following equation:

$$\rho_S C_S \left[\frac{\partial}{\partial t} \mathrm{T}(\mathbf{r},t) + \nabla \cdot (T(\mathbf{r},t)\mathbf{v}(\mathbf{r} \cdot t))\right] - \kappa_S \nabla^2 T(\mathbf{r},t) = 0 \tag{29}$$

Where $\mathbf{v}(\mathbf{r},t)$ is the surrounding fluid velocity and $\nabla \cdot (T(\mathbf{r},t)\mathbf{v}(\mathbf{r},t))$ is a convective term. k_s is the thermal conductivity of water, ρ_s is the density of water, and C_s is the specific heat capacity of water at constant pressure.

The water density surrounding the colloidal collectives will decrease as the temperature of the collectives rises, thus generating an upward convective flow. Due to fluidic continuity, the fluid in other locations will flow toward the center of mass

reduction in a toroidal shape. The Navier–Stokes equation describes this profile of the fluid velocity:

$$\frac{\partial}{\partial t}\mathbf{v}(\mathbf{r},t) + (\mathbf{v}(\mathbf{r},t)\cdot\nabla)\mathbf{v}(\mathbf{r},t) = \mu_S S\nabla^2\mathbf{v}(\mathbf{r},t) + \mathbf{f}_t(T(\mathbf{r},t)) \tag{30}$$

Where μ_S is the surrounding fluid viscosity and $\mathbf{f}_t$ is the force per unit mass due to the temperature gradient. To reduce the computational complexity when solving non-isothermal flow problems, Boussinesq approximation is usually implemented, which implies that the flow density variation is only important in the term $\mathbf{f}_t$, and have no effect in other terms. Typically, in natural convection, $\mathbf{f}_t$ is temperature-dependent buoyancy force, one can estimate $\mathbf{f}_t$ as

$$\mathbf{f}_t(T) = \beta_S g(T(\mathbf{r},t) - T0)\mathbf{z} \tag{31}$$

where β_S is the dilatation coefficient of the fluid, g is the gravitational acceleration, $T0$ is the initial temperature, and $\mathbf{z}$ is the upward z-direction unit vector.

We chose to solve numerically in parallel equations 29, 30, and 31 using COMSOL Multiphysics, a commercial software based on finite element calculations and suited to address problems coupling several different differential equations. Due to the axial symmetry of the system investigated, a two-dimensional model was considered. The Paradiso solver was used on a free triangular mesh. The heat transfer (ht) and laminar flow (spf) modules were used to solve for transient values of coupled fields. The physical parameters of water were taken from the COMSOL library, and their dependence on temperature was taken into account. No slip boundaries are considered and set as thermal insulation. The diameter of the colloidal collective is set at 4 mm and 0.3 mm from the lower boundary of the flow field. The length and height of the tank are set to 30 mm and 10 mm, respectively. The parameters of water in COMSOL are set as follows:

Parameters	Function	Temperature range
Constant pressure heat capacity (Cp)	$12010.1471-80.4072879*T+0.309866854*T^2-5.38186884E^{-4}*T^3+3.62536437E^{-7}*T^4$	[273.15 K, 553.75 K]
Thermal conductivity (k)	$-0.869083936+0.00894880345*T-1.58366345E^{-5}*T^2+7.97543259E^{-9}*T^3$	[273.15 K, 553.75 K]
Coefficient of thermal expansion (β)	$\frac{-1}{\rho(\mathrm{T})}\frac{\partial}{\partial T}\rho(\mathrm{T})$	[293.15 K, 373.15 K]
Density (ρ)	$0.000063092789034*T^3-0.060367639882855*T^2+18.9229382407066*T-950.704055329848$	[273.15 K, 293.15 K]
	$0.000010335053319*T^3-0.013395065634452*T^2+4.969288832655160*T+432.257114008512$	[293.15 K, 373.15 K]
Dynamic viscosity (μ)	$1.3799566804-0.021224019151*T+1.3604562827E^{-4}*T^2-4.6454090319E-7*T^3+8.9042735735E^{-10}*T^4-9.0790692686E^{-13}*T^5+3.8457331488E^{-16}*T^6$	[273.15 K, 413.15 K]
	$0.00401235783-2.10746715E^{-5}*T+3.85772275E^{-8}*T^2-2.39730284E^{-11}*T^3$	[413.15 K, 553.75 K]

Supplementary Table 4 | Parameter settings. Thermal expansion coefficient, dynamic viscosity, liquid density, constant pressure heat capacity and thermal conductivity of water as a function of temperature.

When the temperature difference (*ΔT*) is set as 10 K, 20 K, 30 K, 40 K and 50 K, respectively, the temperature distribution and floating velocity distribution of the whole tank within 1 s are shown in **Supplementary Figs. 9** and **10**, respectively. At 0.25 s, the high-temperature and high-floating-speed zone are mainly concentrated

near the colloidal collectives. The colloidal collectives then transfer heat to the surrounding water, causing the water's liquid density to decrease as the water's

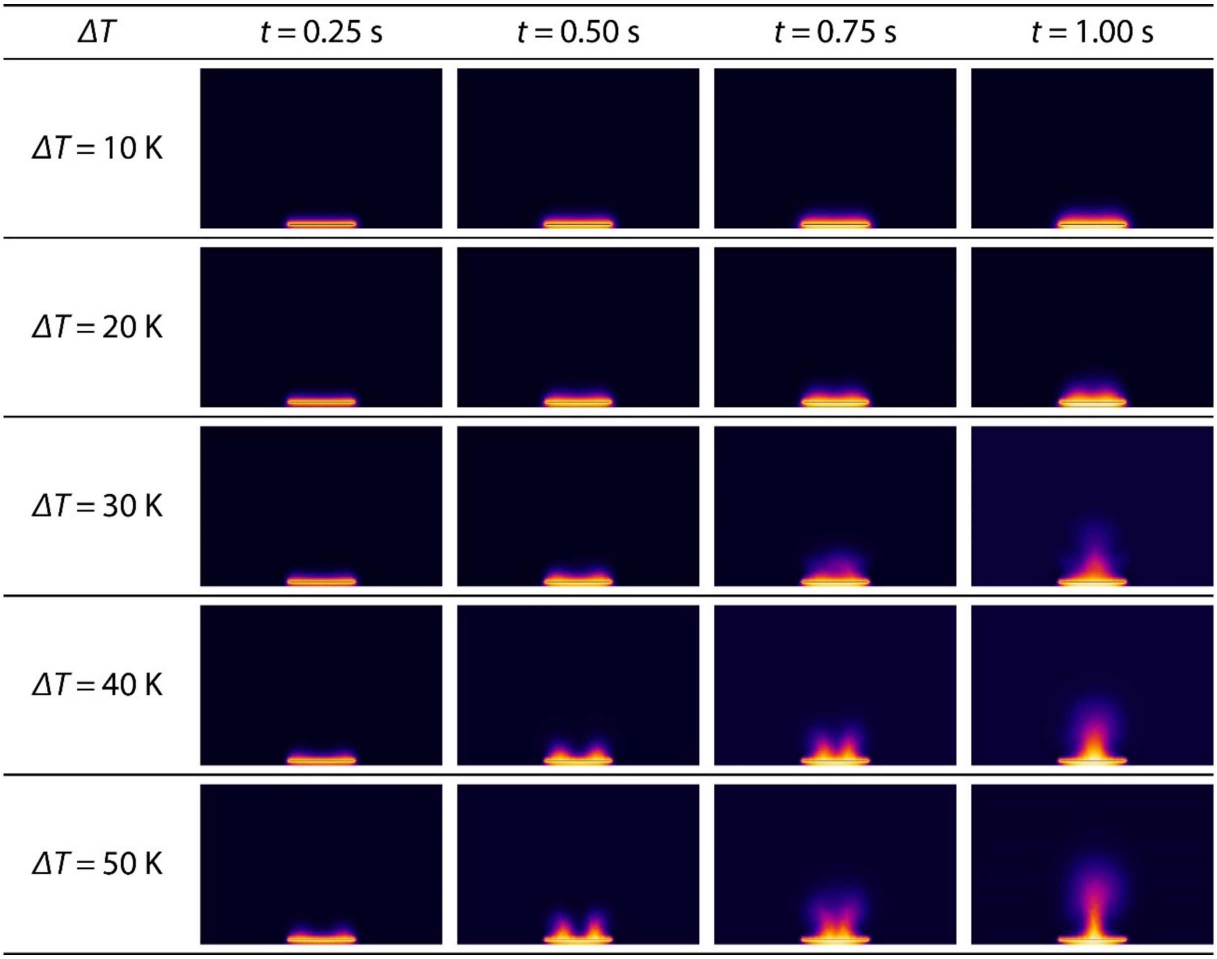

Supplementary Figure 9 | The simulation results of temperature distribution under temperature difference of 10 K, 20 K, 30 K, 40 K and 50 K.

temperature increases, and then the lower-density water flows upward causing buoyant flow. Due to the different initial temperature difference, the temperature distribution in the tank after 1 s is different, and the heat transfer range of the colloidal collectives within 1 s increases with the increase of the temperature difference. The resulting buoyant fluid rises the greater the distance, and the maximum floating speed after 1 second also increases from 0.97 mm/s (ΔT is 10 K) to 14.6 mm/s (ΔT is 50 K).

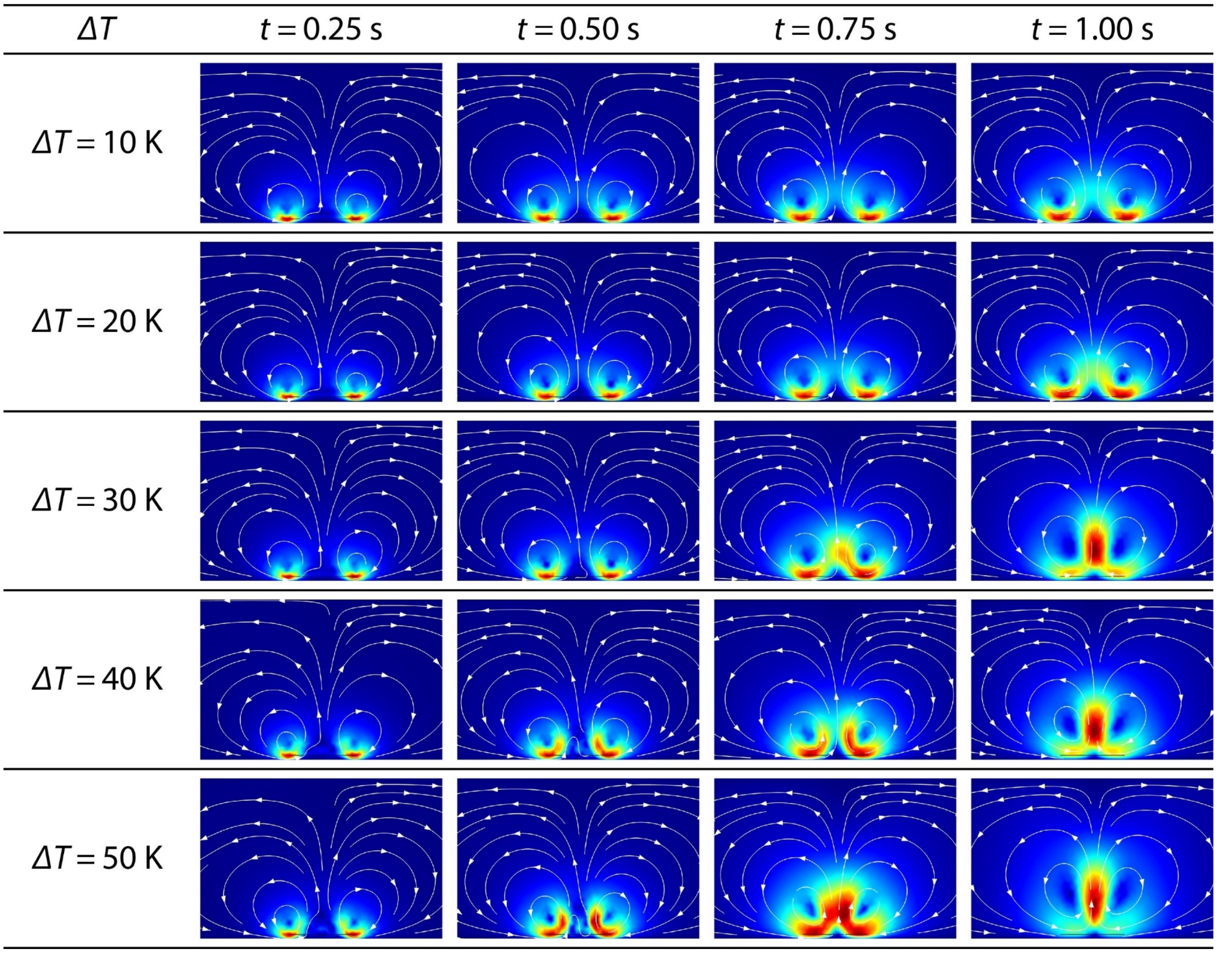

Supplementary Figure 10 | The simulation results of floating velocity distribution under temperature difference of 10K, 20K, 30K, 40K and 50K.

To clearly show the convective flow, add many tracer particles with a diameter of 200 μm to the tank in advance. Convection appeared after irradiating the colloidal collectives with a near-infrared laser with a power of 2 W for 1 s. At 2 s, the buoyant flow reached the top of the liquid surface. The experimental results are consistent with the simulation results (**Supplementary Fig. 11a**). Then, the colloidal collective is driven to float up under the action of the flowing liquid and reaches the top of the liquid surface after 1.8 s. Then the NIR laser beam is turned off, and the colloidal collective cools down. The weakening of the buoyant flow is insufficient to drive the colloidal collective. Finally, the colloidal collective will slowly sink

(**Supplementary Fig. 11b**). It is worth noting that under the action of the rotating magnetic field, the magnetic dipole force between the colloidal particles will make the colloidal collective maintains a complete whole without dispersion. If the magnetic field is turned off during the fall, the colloidal collective begins to disperse, and the dispersed colloidal particles can form colloidal aggregates again (**Supplementary Fig. 11c**).

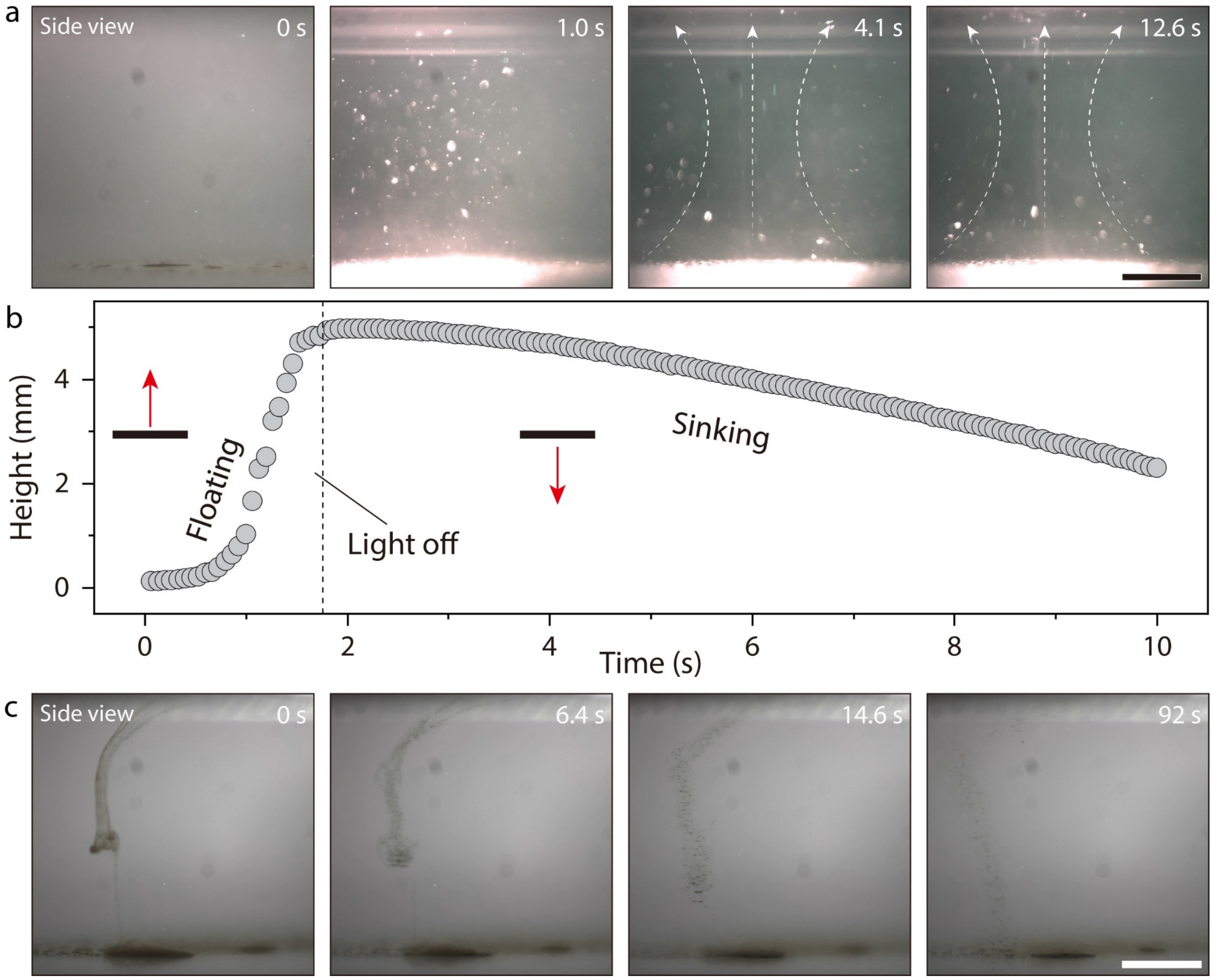

Supplementary Figure 11 | Floating movement of the colloidal collective. a, A convective flow generates as the colloidal collective is heating up under the irradiation of 2 W NIR laser beam. The tracer particles move from the bottom to the top of liquid under the action of liquid flow. **b,** Time-dependent height of a colloidal

collective driven by the convective flow. **c,** Dispersed colloidal collective reassembles to form an entity under the rotating magnetic field.

Furthermore, according to the Navier–Stokes equations, as the laser power increases, the colloidal collective will absorb more light energy, converting it into more heat and thus inducing a greater rate of buoyant flow. Theoretically, the floating speed of the colloidal collective is also faster and is positively correlated with the laser power. We have calculated the average temperature versus time for the entire tank interval and the maximum buoyant flow rate generated versus time by simulation (**Supplementary Figs. 12a and 12b**). The simulation results show that the average temperature of the entire tank area is positively correlated with the temperature of the colloidal collective itself. In turn, the maximum velocity of the buoyant flow it induces becomes larger as the temperature of the colloidal collective increases. To further verify this, we characterized the floating speed of the same colloidal collective at different laser powers (**Supplementary Fig. 12c**). As the laser power increases from 1 to 8 W, the floating velocity of the collective increases from 0 mm/s to 2.8 mm/s.

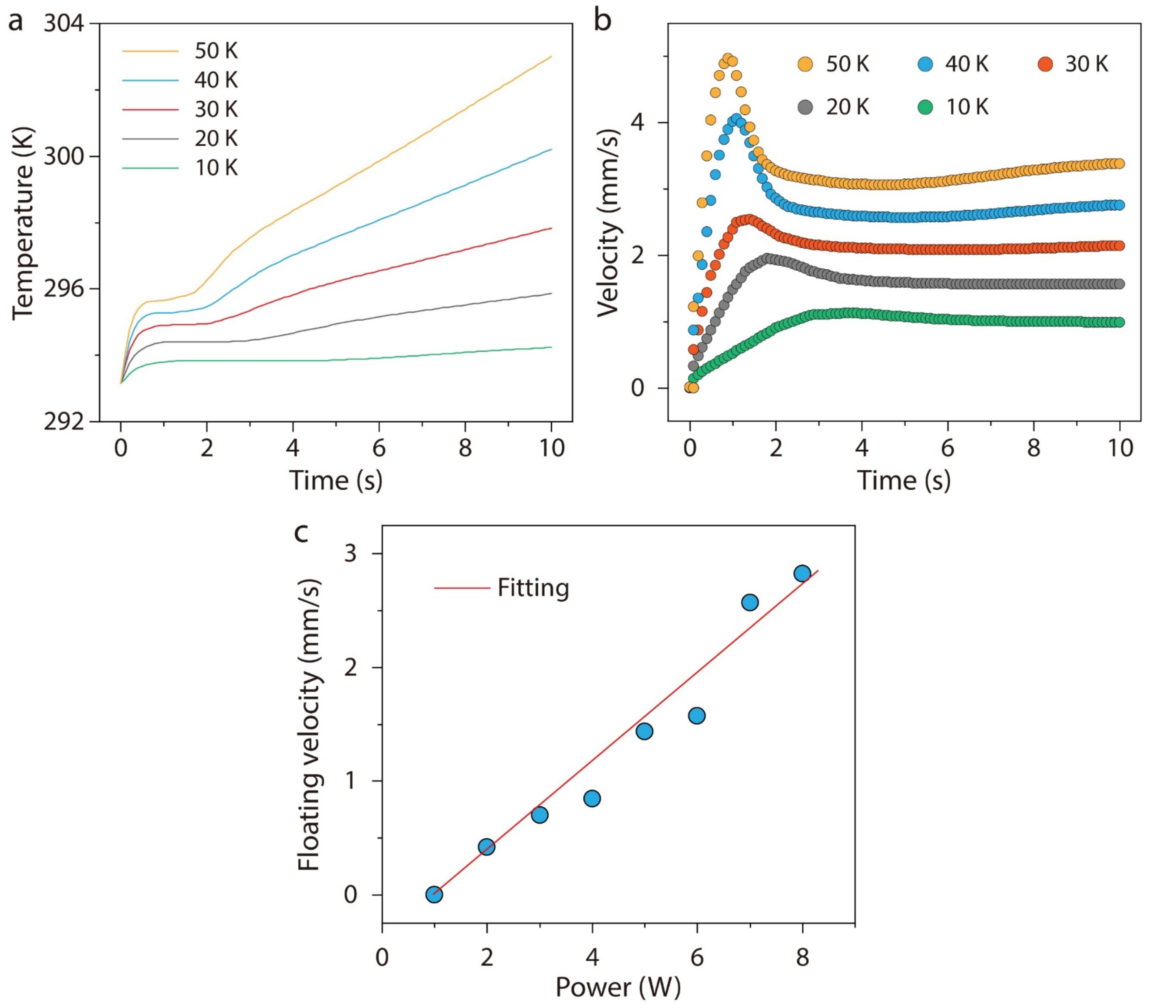

Supplementary Figure 12 | Simulation and experimental results of colloidal collectives rising under convection. a, The average temperature of the whole liquid under different temperature difference. **b**, Maximum flow velocity at different times under different temperature difference. **c,** Floating speed of colloidal collectives under different laser power.

Supplementary Note 8. Fragmentation analysis on colloidal collectives during floating up

In the process of floating of the colloidal collective, it is mainly subject to the buoyancy, drag force and self-gravity, and can be expressed as the following:

$$m\boldsymbol{a} = \boldsymbol{F}_{hydrodynamic} + \boldsymbol{F}_{gravity} + \boldsymbol{F}_{buoyancy} \tag{32}$$

Where m is the mass of colloidal collective, and **a** is the acceleration of colloidal collective.

$$\boldsymbol{F}_{buoyancy} = \mathbf{f}_{\mathrm{t}}(T(\mathbf{r}, t)) \tag{33}$$

However, the buoyancy force depends on the temperature and time at a specific spatial location. Due to the size of the colloidal collectives, the overall buoyancy force is not uniformly distributed during its floating process. This non-uniform distribution can result in possible fractures of the colloidal collectives. The simulation results indicate that the colloidal collective is not subjected to the same buoyancy flow velocity at different spatial locations (see **Supplementary Fig. 13a**). The colloidal collectives consist of colloidal particles that are magnetically coupled to each other by magnetic dipole forces. If the uneven distribution of buoyancy forces varies greatly, it can cause dislocations between the coupled colloidal particles during the rise of the colloidal collective, leading to the fracture of the colloidal collective. In the simulation, the size of the colloidal collective is set to 4 mm, and the temperature difference is set to 10 K. The velocity of the buoyant flow generated in the body of the colloidal collective at 0.5 mm and 1 mm from its body is not uniformly distributed in the 0-2 s range. **Supplementary Fig. 13b** shows the mechanics between two colloidal particles subjected to buoyancy forces, drag forces, magnetic dipole forces, gravity, and bulk forces during the floating process.

$$\boldsymbol{F}_{hydrodynamic} + \boldsymbol{F}_{gravity} + \boldsymbol{F}_{buoyancy} + \boldsymbol{F}_{dipole} + \boldsymbol{F}_{repulsive} = 0 \tag{34}$$

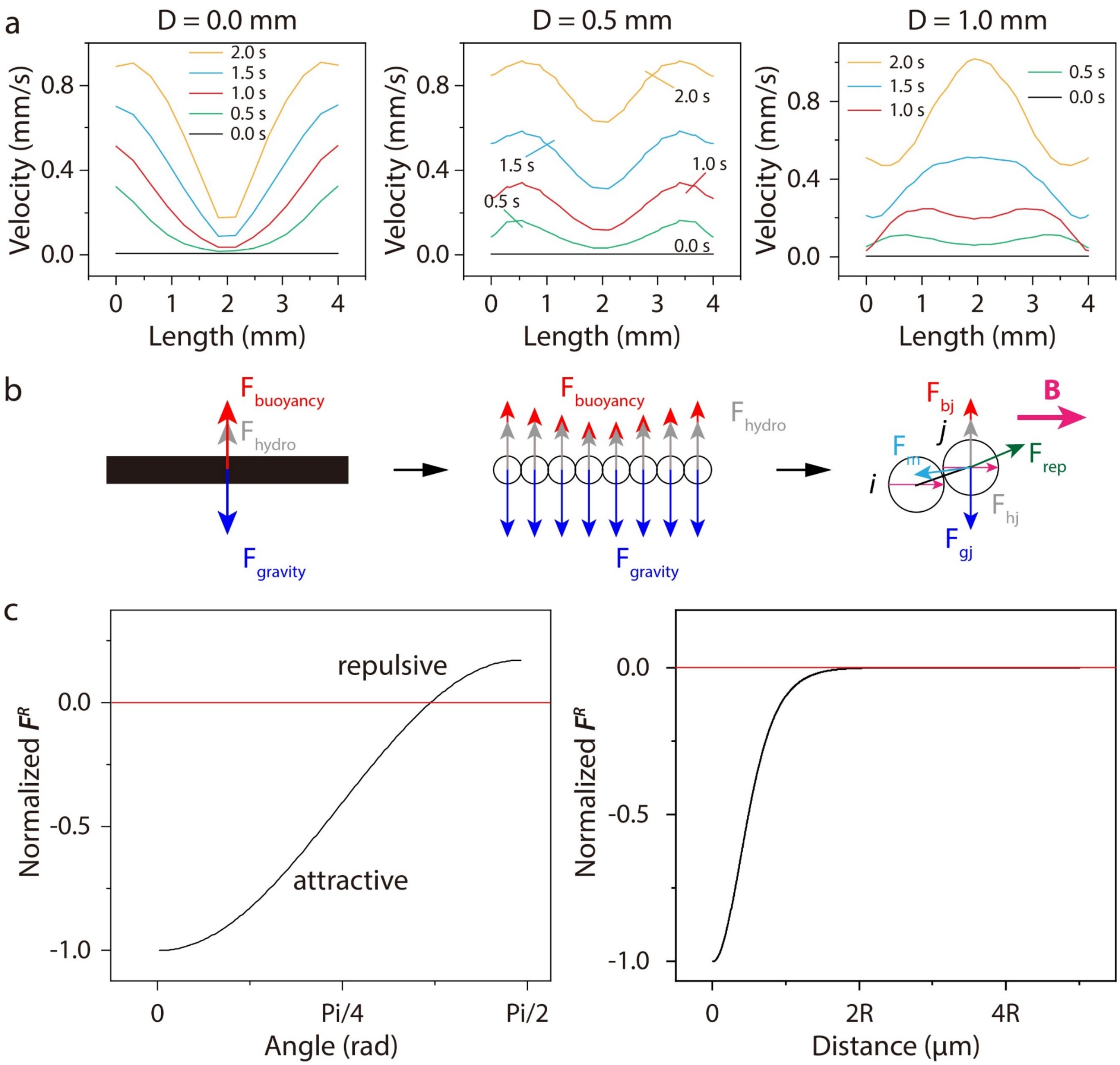

Supplementary Figure 13 | Fragmentation analysis during the rise of colloidal collectives. a, Distribution of the convective flow induced by colloidal collectives was studied for velocities on cut-off lines at heights of 0, 0.5 and 1 mm from it, respectively. **b,** Schematic diagram shows that the stability of the colloidal collective depends on the magnetic coupling between magnetic particles. **c**, The magnetic dipole force between two magnetic dipoles in relation to the angle and relative position.

Based on the two-coupling colloidal particles model, we have determined that magnetic dipole forces between the particles are responsible for maintaining the stability of the colloidal collectives and preventing division during the rise. To further understand this phenomenon, we examined the changes in magnetic dipole force as the relative position and angle of the two colloidal particles changed. **Supplementary Fig. 13c** demonstrates that the magnetic dipole force is strongest when the relative angle between the two particles is 0°. However, when the angle between the two exceeds 60°, the magnetic dipole force between them becomes extremely weak and no longer functions. Similarly, once the relative position of the colloidal particles surpasses the position of two radii, the magnetic dipole force is no longer effective (**Supplementary Fig. 13c**). If this occurs, the colloidal collectives will split.

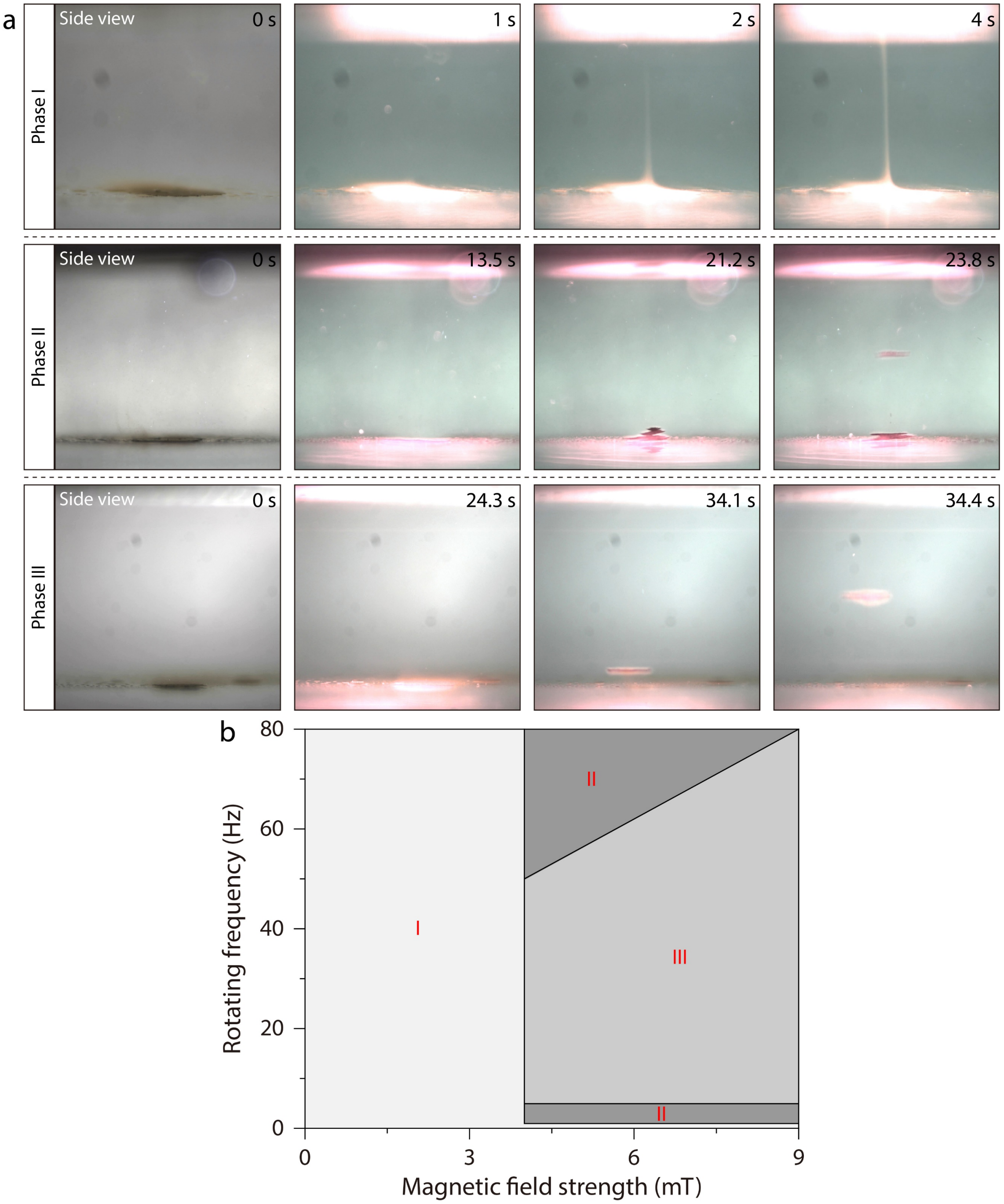

Supplementary Figure 14 | Floating colloidal collectives. a, Different stages of colloidal collectives' uplift. **b**, Phase diagram showing the floating colloidal collectives generated by magnetic fields with different combinations of frequency and magnetic field strength. The corresponding phases of the floating colloidal collectives are represented by I, II, and III, respectively.

By applying rotating magnetic fields with varying strengths and frequencies, we investigated the conditions for the successful formation and upward movement of colloidal collectives (**Supplementary Figs. 14a** and **14b**). It was found that when the magnetic field strength is low (less than 4 mT), colloidal particles have difficulty forming colloidal clusters, regardless of the frequency. Consequently, in the case of NIR light, colloidal particles rise in a dispersed form, as Phase I suggests. When the magnetic field strength increases beyond 4 mT, and the frequency is low (less than 1 Hz), the colloidal particles form a longer chain structure, which is more dispersed from each other and does not form a monolithic structure. As a result, the buoyancy force generated during light easily breaks the colloidal chain structure and causes them to rise in a dispersed mode during the uplifting process (Phase I).

As the frequency increases, the long colloidal chain structure begins to break and form shorter chains (2-5 Hz), and most of the shorter chains begin to coalesce and form a cohesive unit. However, the fluid forces induced by the colloidal short chains are so large that the structure within the colloidal collective is not stable. Therefore, under NIR laser irradiation, the colloidal collective disperses into multiple colloidal clusters to rise.

When the magnetic field input frequency increases to 5-50 Hz, the short chains of the colloids begin to collapse to form a dense structure, causing the colloidal collectives to form colloidal crystals. Then the buoyant flow created by the photothermal conversion causes it to float up. For higher magnetic field strengths, this frequency range is greater. However, as the frequency increases, the entire magnetic field device develops a greater susceptibility, leading to a decrease in the overall magnetic field strength. Consequently, under NIR laser irradiation, the colloidal collectives disperse to form several clusters and then float upwards.

Supplementary Note 9. The motion mechanism of colloidal collectives underwater

Colloidal collectives can achieve vertical upward motion by generating a buoyant flow through photothermal conversion. This is because the buoyancy flow has a 360° symmetry, and the colloidal collectives are located precisely at the center of the buoyancy flow, which drives them upwards vertically. As shown in **Supplementary Fig. 15a**, by pulsed vertical irradiation, the colloidal collective rises vertically. And hover at the target position caused by the buoyant flow, with a floating d range within 1 mm. The optical spot can selectively shine on one side of the colloidal collectives. In that case, the overall temperature distribution is not uniform, and the induced buoyancy flow will then drive the colloidal collectives horizontally. The colloidal collectives can be continuously moved in one direction by intermittent irradiation. **Supplementary Fig. 15b** compares the patterns of buoyancy flow generated by the three modes of colloidal collectives. When the colloidal collective is uniformly irradiated in the optical field, the fluid velocity distribution over the colloidal collective is homogeneous, and the buoyant flow it induces is symmetrical all the time so that it rises vertically. When the colloidal collective is irradiated purely on the left side, the temperature on the left side is higher than on the right side, so the induced buoyancy flow drives it to the left. When the colloidal collective is irradiated to the right, the right side will be warmer than the left side, thus inducing a buoyant flow that will drive it to the right. Our experiments further verify the controllability of the orientation of colloidal collectives in water. When the laser is raised on the side of the colloidal collective, the collective can move left and right, respectively (**Supplementary Figs. 15c** and **15d**). The experimental results show the relationship between horizontal movement distance and time during translation.

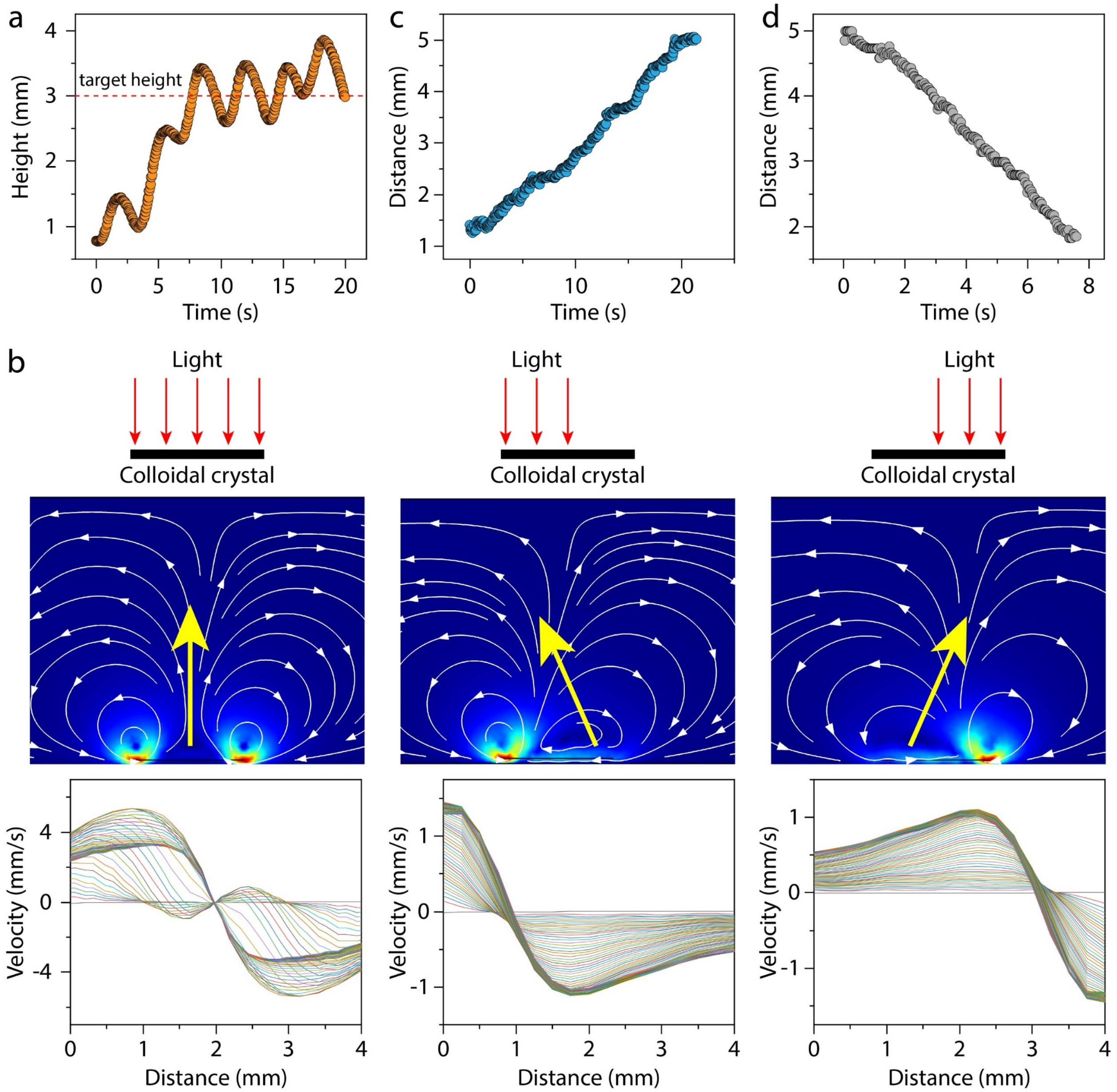

Supplementary Figure 15 | Colloidal collectives perform directional motion underwater. a, Relationship between the height and time when the colloid collectives are hovering. **b,** Simulation results of the fluid fields generated when optical spots are irradiated at different positions on the colloidal collectives. Relationship between distance and time in the horizontal direction when the colloidal collective is moving from left to right (**c**), and moving from right to left (**d**).

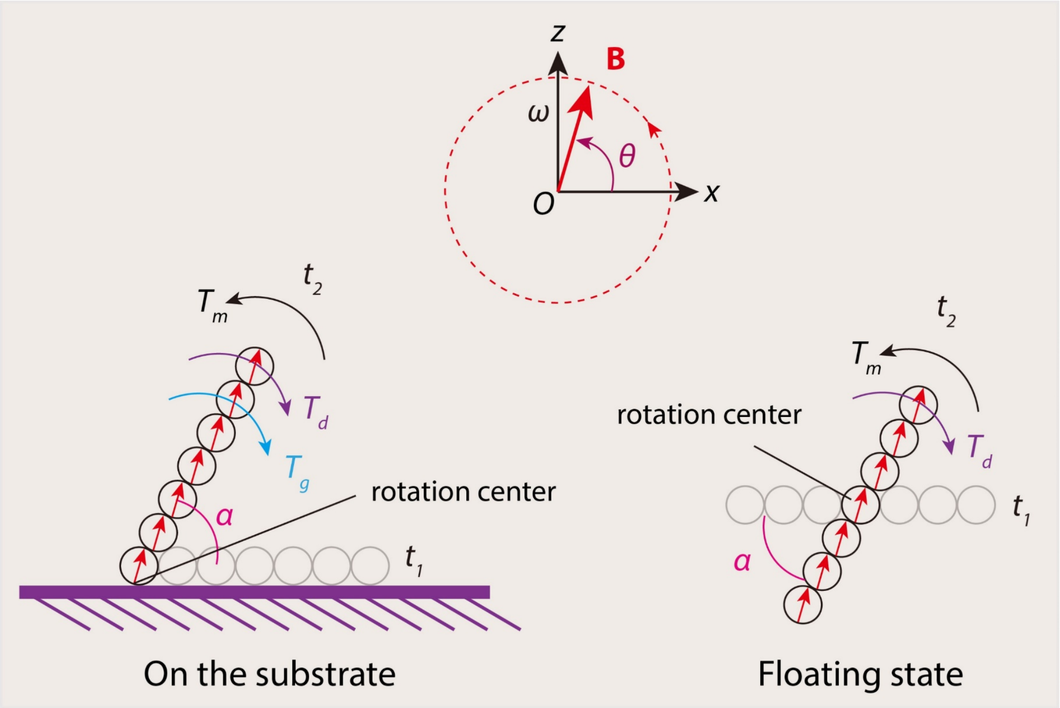

Supplementary Figure 16 | Contrast analysis of colloidal collectives' rotation process (on substrate and floating state).

In addition, when the colloidal collectives fall, the magnetic field can also make the colloid overall through the flip motion (along the *x*-axis and the *y*-axis). It is worth noting that the flip movement of colloid collectives during the falling process is not easy to produce division. This is mainly because when the colloidal collectives are flipped along the bottom surface, the rotation center is at the end of the colloid collectives; the rotation center is in the center of the colloid collectives when the colloid collectives are flipped during the falling. Compared with the flip motion of the colloidal collectives on the bottom surface, the viscosity resistance of the colloidal collectives is small during the falling (about 0.5 times the flip motion on the bottom surface), and it is not affected by the role of gravity torque. Therefore, the magnetic driving torque of the colloidal collectives can easily overcome the effect of the sticky resistance torque, which is hard to split.

Supplementary Note 10. Analysis on water-to-air and air-to-water transitions of colloidal collectives

We establish the analysis models for colloid collectives when they exit from and enter in the water (**Supplementary Fig. 17a**). The colloidal collectives are mainly subject to the surface tension F_S, buoyancy F_B, and its own gravity F_G. When it is irradiated with NIR laser, it will also receive hydrodynamic force F_H from the flowing fluid. The surface tension force F_S: since σ can also be interpreted as force per length,

$$F_S = \sigma \cos\gamma \, (2\pi a) \tag{35}$$

The buoyant force F_B:

$$F_B = \rho_f g \pi a^2 (\frac{H}{2} - h) \tag{36}$$

The gravity F_G:

$$F_G = \rho_c g \pi a^2 H \tag{37}$$

The hydrodynamic force F_H:

$$F_H = \frac{1}{2}\rho_f u^2 D \pi a^2 \tag{38}$$

$$F_B + F_H - F_S - F_G = 0 \tag{39}$$

Where σ is the surface tension coefficient along the fluid interface, γ is the contact angle, a is the radius of the colloidal collectives, ρ_f is the density of the surrounding fluid, g is the gravity force per unit mass, H is the height of the colloidal collectives, h is the displacement from the center of the colloidal collectives to the reference fluid level, ρ_c is the density of the colloidal collectives, u is the flow speed of induced buoyant flow, and D is the drag coefficient.

For colloidal collectives to achieve water exit, a problem must be solved first, which is to overcome the surface tension of the water. When the colloidal collective reaches the water-air interface, it will be hindered by the surface tension. At this time, the continuous high-intensity optical field will cause the induced convection to be greater. Therefore, the hydrodynamic force enables the colloidal collectives to break the water surface tension. However, it can be found that the colloidal collectives will be ruptured into many fragments when crossing the water, and then regather under the rotating magnetic field (**Supplementary Fig. 17b**). This is mainly due to the uneven force that the colloidal collective is suffered during crossing. The magnetic dipole forces inside the colloid are not enough to maintain the stability, and it will eventually break into multiple small colloidal collectives. In the initial state, the colloid collective is on the bottom of the water. After laser irradiation, the colloid collective floats to the below the water-air interface. With the continuous irradiation of NIR laser, the colloidal collective begins to split. As the colloidal collective is divided, the colloid collective crosses break the water surface. Then driven by the rotating magnetic field, the colloidal collective fragments begin to regather to form a complete colloidal collective.

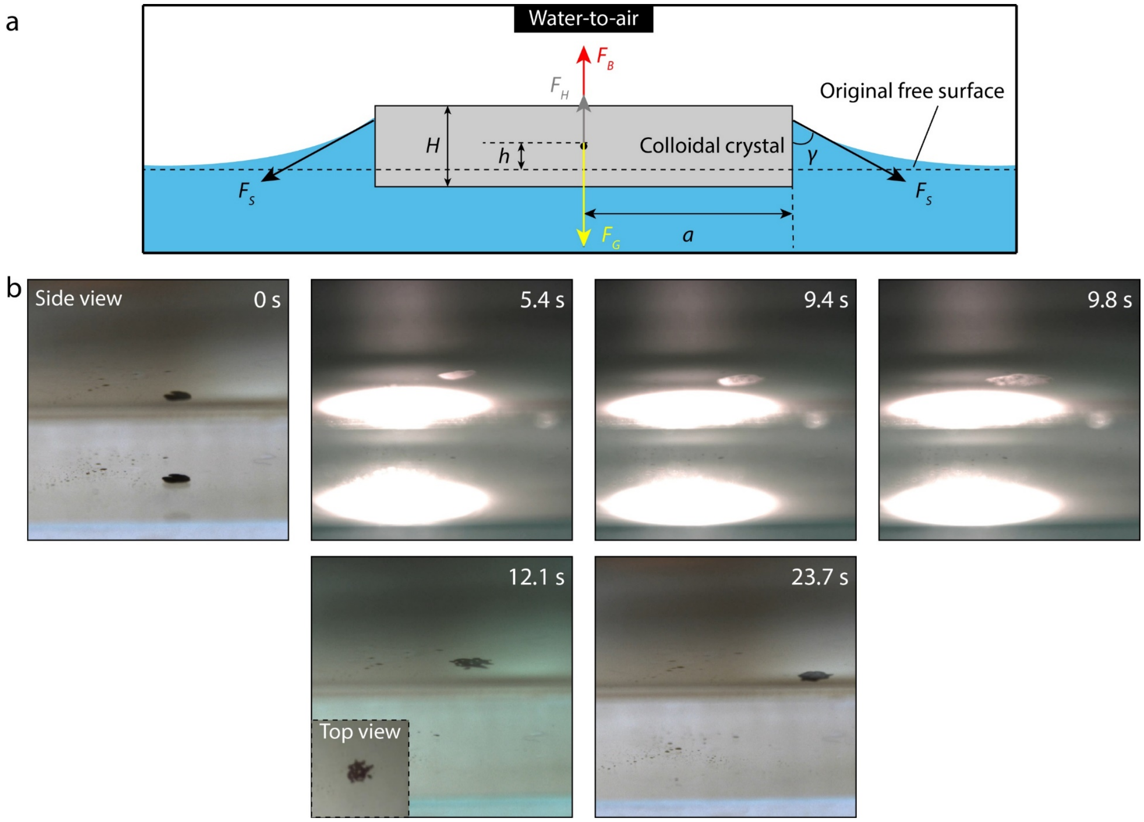

Supplementary Figure 17 | The dynamic model and experimental results of the colloidal collectives crossing the water-air interface. a, Dynamic model of the colloidal collective transits from underwater to the water surface. **b,** Experimental results of the colloidal collective crossing the water-air interface.

When the colloidal collective reaches the water surface, close the NIR laser. Colloidal collectives are no longer affected by flowing fluid F_H, only gravity F_G, buoyancy F_{B1}, and surface tension F_{S1}:

$$F_{S1} = \sigma\, cos\gamma_1\, (2\pi a) \tag{40}$$

The buoyant force F_B:

$$F_{B1} = \rho_f g \pi a^2 (\frac{H}{2} + h) \tag{41}$$

The gravity F_G:

$$F_G = \rho_c g \pi a^2 H \tag{42}$$

The hydrodynamic force F_H:

$$F_H = 0 \tag{43}$$

$$F_{B1} + F_{S1} - F_G = 0 \tag{44}$$

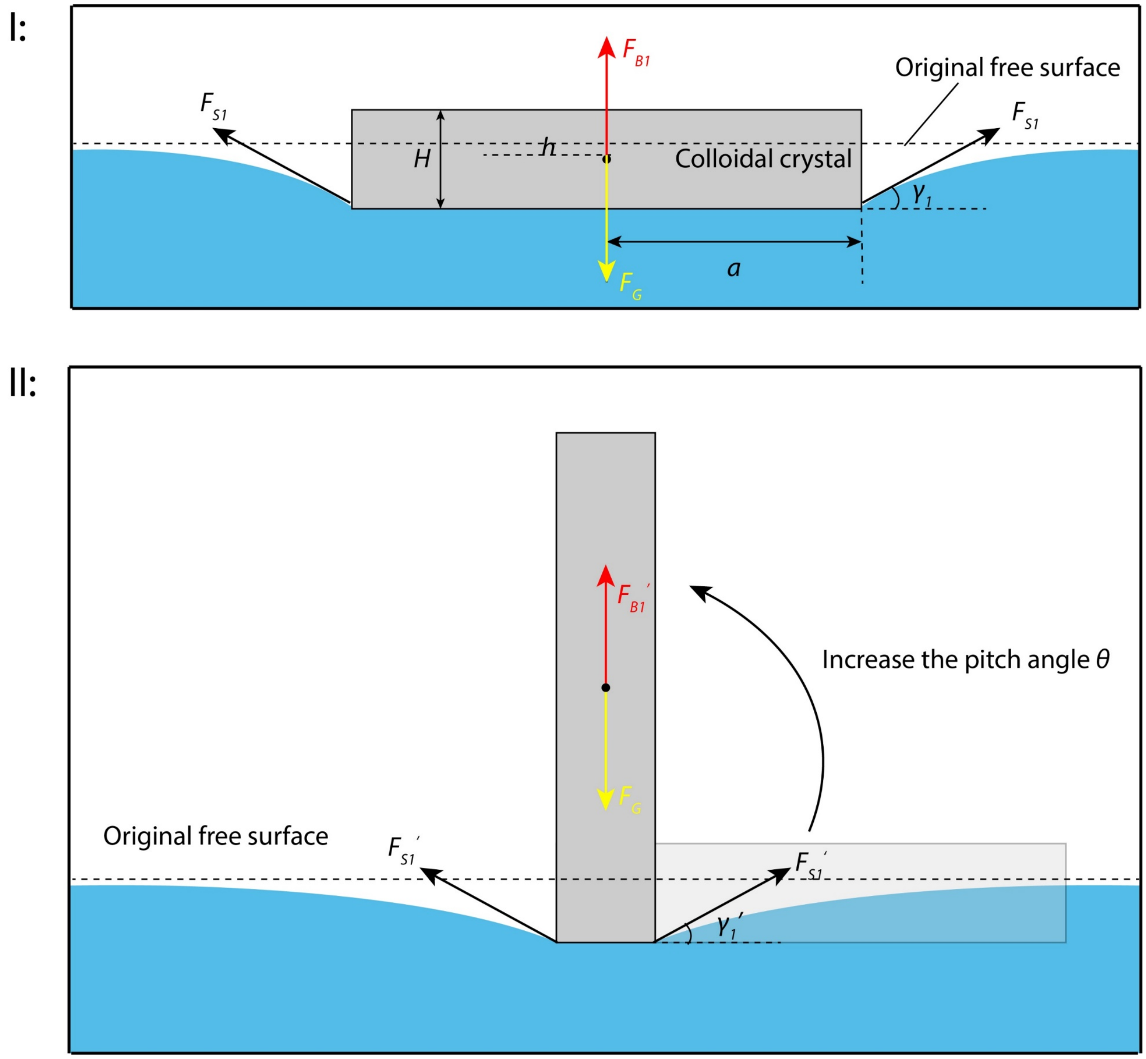

Supplementary Figure 18 | Analytical diagram of the strategy of colloidal collectives sinking into water.

In order to realize the sinking of the colloidal collectives, the surface tension and buoyancy need to be reduced. Here, by increasing the angle between the rotating magnetic field and the horizontal plane, that is, the pitch angle, then drives the collective to flip on the water surface. The net contact length of the flipped colloidal collective is greatly reduced. When we focus on an extreme state, when the collectives are flipped to 90° (the experiment shows that the collectives will sink into the water when they are flipped to 20°). As shown in the **Supplementary Fig. 18**, in the vertical state of the collective, the net contact length with the water surface changes from the original 2a to H, which greatly reduces the surface tension. In addition, due to the adjustment of the colloidal collectives' posture, the buoyancy it receives is reduced. Under the influence of gravity, the collectives sink into the bottom.

Supplementary Note 11. Locomotion on the water surface driven by magnetic field

In addition to the controllable transition from water to air and air to water, the colloidal collectives can also achieve controllable locomotion on the water surface. As shown in the **Supplementary Fig. 19**, by applying an inclined rotating magnetic field, that is, the pitch angle is generally less than 10°, and the colloidal collective can perform controllable motion on the air-water interface. The forces acting on colloidal collectives include the form drag, buoyancy, added inertia, viscous drag, surface tension force and Marangoni force, which can be estimated by

$$|F| \sim \rho U^2 A + \rho g h A + \rho V \frac{dU}{dt} + \mu U \frac{A}{w} + \gamma \frac{A}{w} - \nabla \gamma A \tag{45}$$

Where ρ is the density of water, g is the gravitational constant, μ is the viscosity of water, U is the speed of the body, V is the characteristic volume of the body in consideration, A is the characteristic area of the body, w is the characteristic width of the body, h is the depth of the body from the original water surface, and γ is the surface tension coefficient. The first term is the form drag. The second term, which is the buoyancy term, is mostly vertical but may contain a horizontal component. The third term is the added inertia term. The fourth term is the viscosity drag. The fifth term, which corresponds to the surface tension force, that may also have a horizontal component. the sixth term, called the Marangoni force. The relative magnitudes of the six forces enumerated in Equation 45 are prescribed by five dimensionless groups, the Reynolds *Re*, Weber *We*, Bond *Bo*, Strouhal *St*, and Marangoni *Ma* numbers, defined, respectively, by

$$Re = \frac{Uw}{\nu} \tag{46}$$

$$We = \frac{\rho U^2 w}{\gamma} \tag{47}$$

$$Bo = \frac{\rho g h}{\gamma / w} \tag{48}$$

$$St = \frac{f w}{U} \tag{49}$$

$$Ma = \frac{\nabla \gamma}{\gamma / w} \tag{50}$$

Assessment of the magnitudes of these dimensionless groups indicates that the great majority of water walkers depend principally on some combination of curvature forces and form drag for their forward propulsion. For colloidal collectives, because the rotating magnetic field changes the surface tension, it can no longer be balanced in the horizontal direction, which can cause directional motion. When the tilt direction of the rotating magnetic field is adjusted, the collectives can also achieve controllable locomotion on the water surface.

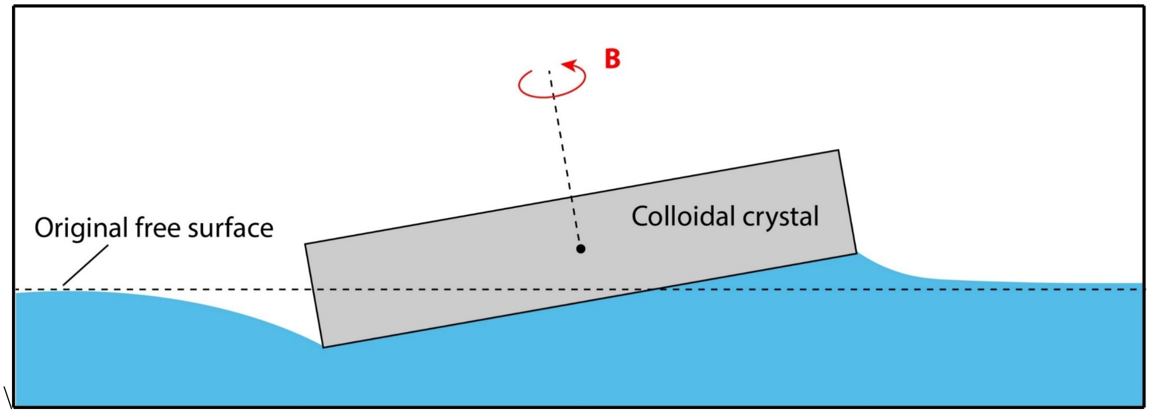

Supplementary Figure 19 | Side view of colloidal colectives actuated by the inclined rotating magnetic field.

Supplementary Note 12. Locomotion on the water surface driven by NIR light

From equation 45, the Marangoni force can also be applied to drive the floating object on the water's surface. Therefore, in addition to the rotating magnetic field driving colloidal collectives for motion, the photothermal surface tension effect can also directly achieve the directional movement of the colloidal collectives. A liquid with a high surface tension pulls more strongly on the surrounding liquid than one with a low surface tension (Marangoni effect). Based on this principle, the optical field has been used to generate thermal surface tension gradients at desired positions and cause the colloidal collectives to flow away from regions of low surface tension (**Supplementary Fig. 20a**). Generally, when a light beam is directly irradiated onto liquids, surface tension gradients are formed by local photothermal heating. Therefore, a laser or focused sunlight can selectively generate thermal surface tension gradients around a floating object without applying toxic or exhaustible chemical intermediates for actuation. The localized heating can create a surface tension gradient, leading to linear motions of the colloidal collectives. As shown in **Supplementary Figs. 20b** and **20c**, the simulation results of the COMSOL Multiphysics show that when the temperature gradient on both sides of the colloidal collectives, it will induce the Marangoni flow, and the direction of the fluid flow will from high temperature to low-temperature areas. As shown in **Supplementary Fig. 20d**, under the NIR laser, the colloidal collectives can perform directional motion on the surface according to the predesigned trajectory.

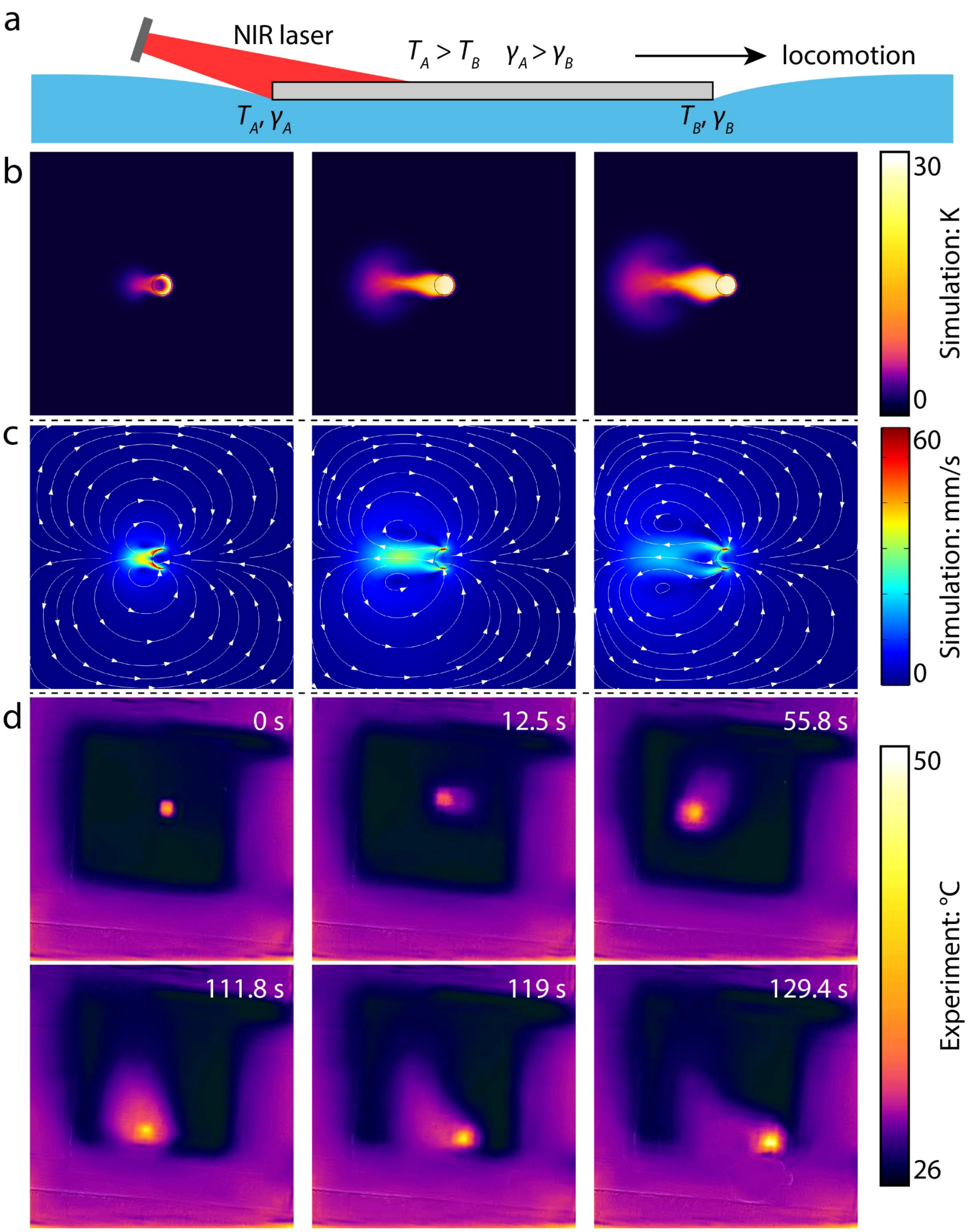

Supplementary Figure 20 | Photothermal surface tension effect. a. Schematic illustrations of the locomotion of colloidal collective induced by the NIR light. **b.** Simulation results of temperature distribution of colloidal collective versus time. **c.** Simulation results of flowing velocity distribution of colloidal collective versus time. **d.** Motion trajectories of the colloidal collective irradiated by the NIR laser.

Supplementary Note 13. Building sophisticated assembled structures using 3D drifting colloidal collectives

While the scenario of manipulating chemical structures one atom at a time, as proposed by Prof. Feynman in 1959, remains in its infancy 50 years later, the manipulation of colloidal spheres with shapes reminiscent of atoms, but a few orders of magnitude larger, has become an active territory for materials scientists. Due to the structural similarities with atomic systems, colloidal assembly has become an important model system for studying the atomic world. Colloidal particles are considered building blocks for materials like atoms are the bricks of molecules, macromolecules, and crystals. Investigating colloidal self-assembly as a means of nanostructure fabrication also holds technological implications. Colloidal structures prepared through self-assembly may find applications in, for example, photonic/plasmonic devices, nanoscale electronics, high-efficiency energy-conversion/energy storage, miniature diagnostic systems, drug/gene delivery, and hierarchically structured catalysts. The primary means of guiding colloidal assembly include organization on a patterned substrate, Langmuir-Blodgett assembly, surfactant-assisted assembly, assembly in nematic liquid crystals, assembly in emulsions or inverse emulsions, assembly in fibers and cells, and external field-guided particle assembly. Among the external field-guided particle assembly approach, magnetic fields can direct magnetic colloidal particles to form chains and dynamically self-assemble into membranes. However, existing magnetic fields are typically applied to 2D structures. It will be another step forward if new particle manipulation techniques are developed to assemble colloids in 3D arrays.

Under the external magnetic field, it is possible to assemble colloidal structures that do not represent the most thermodynamically stable arrangements. Guidance through spatial confinement or by interfaces provides the means for attaining unusual geometries and patterns. As shown in **Supplementary Fig. 21a**, two

colloidal collectives are suspended by stacking them vertically between two vertical walls and keeping them spaced apart, utilizing the ability of the colloidal collectives to move in three dimensions. It is also possible to stack multiple colloidal collectives vertically and vary the structure's height by controlling the optical field's intensity (**Supplementary Fig. 21b**). Suppose these can be locked into place by forming strong interactions. In that case, it becomes possible to achieve molecular analogs of kinetically stabilized structures, including very open structures that have been elusive for colloidal assemblies.

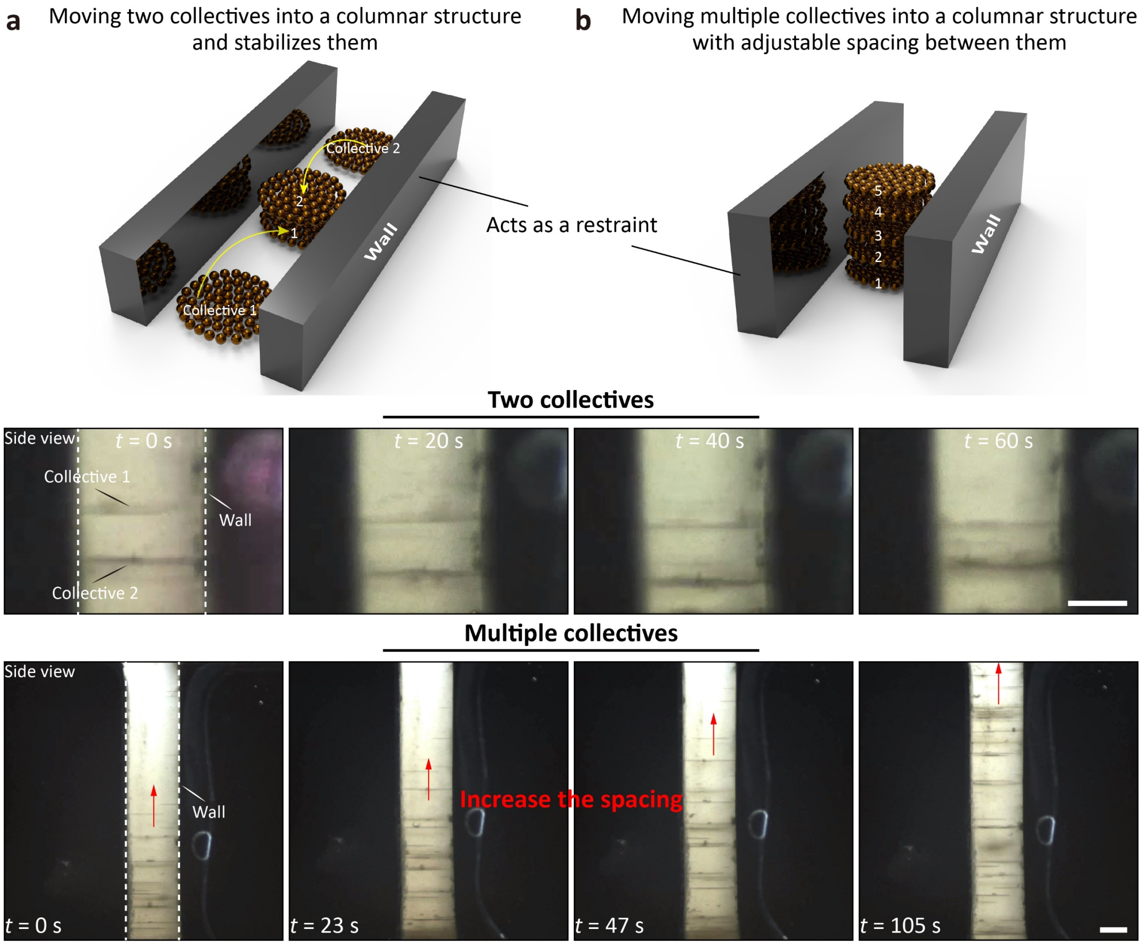

Supplementary Figure 21 | Colloidal collectives as building blocks. A, Schematic shows that two colloidal collectives can be moved to a position of overlap along the axis and kept suspended and stabilized (the experimental diagrams in the first row

below show a side view showing that the two colloidal collectives can be suspended and kept at a certain spacing of about 1.5 mm for 60 s.) **B,** Schematic shows that multiple colloidal collectives can be moved to coincide along the axis and that the spacing between them can be adjusted (the experimental figure in the second row below shows a side view showing the increasing spacing of multiple axially coincident colloidal collectives that can be achieved by gradually increasing the intensity of the light field). Magnetic field (f: 50 Hz, B_m: 9 mT, θ: 0°). Scale bars, 1 mm.

Supplementary Note 14. Environmental adaptability of colloidal collectives

We obtained liquids of different viscosities by mixing different ratios of water and glycerol and studied the motion behavior of colloidal collectives in the liquids. **Supplementary Figure 22a** shows that the uplift motion speed of colloidal collectives decreases as the viscosity of the liquid increases. The colloidal collectives can still move in three dimensions and remain undispersed in a liquid with a viscosity of 80 mPa·s. In addition, **Supplementary Figure 22b** shows that colloidal collectives can move in three dimensions and remain undispersed in biofluids with high ionic concentrations (e.g., fetal bovine serum (FBS) and blood plasma). The FBS and blood plasma contain various ions, including sodium ion (Na^{+}), potassium ion (K^{+}), calcium ion (Ca^{2+}), magnesium ion (Mg^{2+}), chloride ion (Cl^{-}), bicarbonate ion (HCO_3^{-}), phosphate ion (PO_4^{3-}), sulfate ion (SO_4^{2-}). The ionic strength of FBS and plasma are approximately 167 mmol/L and 172 mmol/L, respectively. These experimental results indicate that the proposed method is feasible in an environment rich in ions.

In addition to the various ions, FBS also contains proteins, growth factors, hormones, lipids, and carbohydrates, while plasma contains proteins, glucose, amino acids, lipids, vitamins, urea, creatinine, and various hormones, etc. These components are commonly found in physiological environments and contribute to the complexity of biological fluids. Therefore, the experimental results also demonstrate the adaptability of our magnetic colloidal collective and the bimodal actuation strategy in complex biological fluid environments.

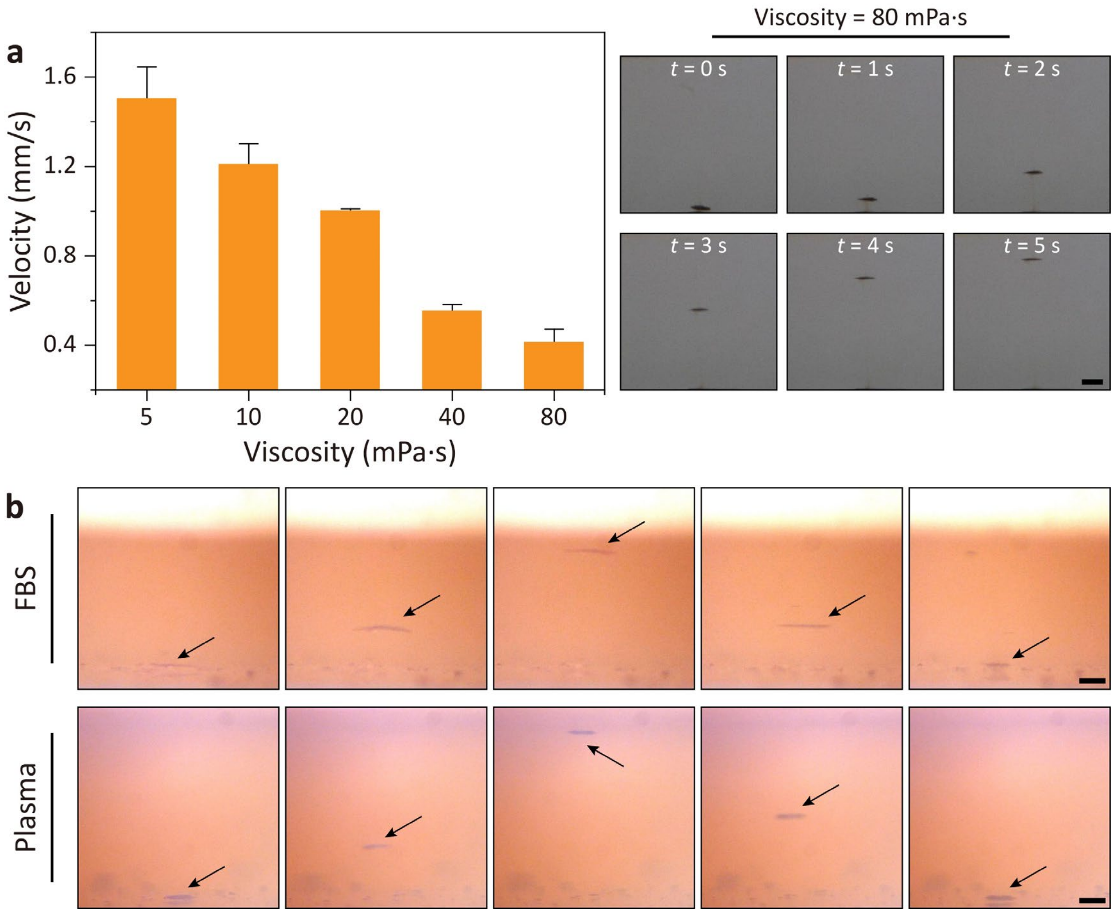

Supplementary Figure 22 | Environmental adaptability of colloidal collectives. **a.** Relationship between the rate of upward motion of colloidal collectives and the viscosity of the liquid. The snapshots show the process of colloidal collective uplift in an 80 mPa·s liquid. **b.** Colloidal collectives move in biofluids with different ionic concentrations. The black arrows indicate the position of the colloidal collective. Scale bars, 2 mm.

Supplementary Movies

Supplementary Video 1. Simulation and experiments of the formation of colloidal collectives

Supplementary Video 2. Gravity-resisting behavior of the colloidal collectives

Supplementary Video 3. Photothermal effect of dispersed colloidal collectives

Supplementary Video 4. Convection drives colloidal collectives to float

Supplementary Video 5. Colloidal collectives perform 3D locomotion underwater

Supplementary Video 6. Tuning the posture of colloidal collectives underwater

Supplementary Video 7. Colloidal collectives exiting the water

Supplementary Video 8. Colloidal collectives entering the water

Supplementary Video 9. Colloidal collectives moving on the water surface

Supplementary Video 10. Adaptive locomotion of colloidal collectives: climbing the wall, passing through the channel, and spanning the gap
